# Low cost rotating disc electrode built using accessible hand tools and 3D printing

**DOI:** 10.1016/j.ohx.2025.e00626

**Published:** 2025-01-31

**Authors:** Adam Shnier, Tarisai Velempini, Anzel Falch

**Affiliations:** Molecular Sciences Institute, School of Chemistry, University of Witwatersrand, Johannesburg 2050, South Africa

**Keywords:** *RDE*, Electrochemistry, Electrolysis, Education, Rotor, Electrode

## Abstract

Rotating disc electrodes (RDEs) are ubiquitous among electrochemistry labs for their versatility. They serve to mitigate the mass transport limitations in experiments through hydrodynamic control. Commercially available RDEs cost thousands of USD ($) making them unaffordable for many lower budget research groups or education institutions. Affordable designs exist in literature to make these, but precision machined parts are required. The presented prototype is fabricated using a 3D printed design and common hand tools, providing clean and reproducible data. This facilitates production in a wider range of environments for research and education applications, as is ideal in the South African context in which it was designed.

## Graphical abstract

**Figure:**
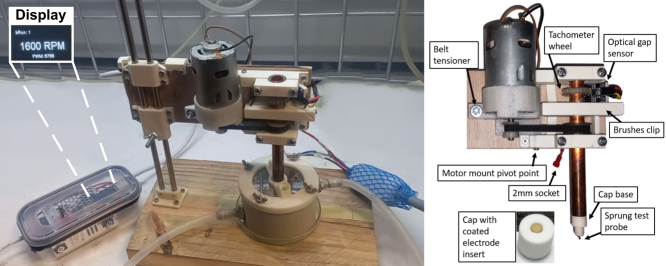

**Table:** 

**Specifications table**	

**Hardware name**	*Belt driven rotating disc electrode*

Subject area	•*Engineering and material science* •*Chemistry and biochemistry* •*Environmental, planetary and agricultural sciences* •*Educational tools and open source alternatives to existing infrastructure* •*General*
Hardware type	•*Electrical engineering and computer science* •*Mechanical engineering and materials science* •*Electrochemistry electrode*
Closest commercial analog	*Pine Research’s WaveVortex 10 Electrode Rotator, Biologic’s BlueRev Rotating Disc Electrode, ALS’s RRDE-3A, Ivium’s rotator apparatus.*
Open source license	Software: MIT Hardware: SHL-2.1
Cost of hardware	<$100 (USD)
Source file repository	Mendeley Data, https://doi.org/10.17632/r4mh958pgm Code: Github, https://github.com/AShnier/RDE

	

## 1 Hardware in context

*Background*

Electrochemistry studies the movement of electrons during redox reactions at a polarised electrode surface. A crucial region in this process is the Nernst diffusion layer, where electron and species exchange occurs. In a static electrolyte, this layer thickness and the species concentrations near the electrode surface have a dependency on diffusion and rate of electron exchange at the polarised electrode surface. The Nernst diffusion layer and these species concentrations will change until a steady state is reached.

Forced convection is a method to control and reduce the size of the diffusion layer; hence enhancing the transfer of species and electrons, this approach is known as hydrodynamic electrochemistry. The use of a rotating disc electrode (RDE) is a classical technique in hydrodynamic electrochemistry utilising forced convection to control convective mass transport [1], [2], [3]. In this method, the working electrode is rotated to create a constant laminar flow of analyte to the electrode’s active surface. This ensures a steady and reproducible mass transport rate while unbound reaction products are removed from the electrode surface [4]. This controlled mass transport allows for the adjustment of kinetically dependent reaction rates thus providing a wide range of reaction time scales between diffusion-controlled and activation-controlled reaction rates. Consequently, it enables the exploration of various kinetic and mechanistic aspects of the reactions under study [1], [5].

*Existing*

Commercial RDEs are available at prices upward of $ 5000, with some suppliers even exceeding the $ 10000 mark. For research or education facilities and groups that have limited budgets and infrastructure; acquisition becomes more difficult, time consuming and sometimes near impossible. When this is coupled with lead times, including inter-continental shipping, the full process has the potential to severely impact student timelines and limit productivity [6]. These consideration can be especially pertinent in developing countries, where there are often significant geographic distance from the equipment manufacturers.

An open hardware RDE has been presented by Whittingham et al. 2022 [7]. This is a well designed rotor which requires only a few specialised processes, which may or may not be available to the targeted niche. Namely, these are dependent on a lathe to shape the main shaft and a dual extruder 3D printer for the electrodes. A further unexpectedly difficult challenge includes acquiring a DC motor that reaches suitable speeds with a built in encoder as suggested by [7]. A list of failed web searches from a South African IP address are included in SI Section 13.

*Our prototype*

The RDE prototype presented requires access to a basic, single filament 3D printer and does not require any lathe work. While our prototype does not constitute a precisely machined shaft, we show it as a suitable, reliable, and repairable tool in electrochemical characterisation for the applications demonstrated.

## 2 Hardware description

The designed rotating disc electrode (RDE) allows the controlled rotation of an electrode in solution at a selectable rotational rate with reliable electrical continuity facilitated between the electrode and a potentiostat. It offers a low cost assembly accessible to those who have access to a 3D printer, an electric drill and hand tools. Notably it does not require access to a metal lathe or a machining shop.

Applications include:

• Electro-catalyst testing, and general electrochemistry.
• Characterisation of electrodes with modified surfaces such as coatings or treatments.
• Teaching pulse width modulation (PWM) based motor control and electrochemistry principles (diffusion layer, mass transport and activation barrier control).
• The RDE is orders of magnitude lower cost than commercial alternatives and is easily repairable with readily available components.
• Stability testing of electrochemical systems where the parallelisation is invaluable for multi-day experiments. This can be achieved through the production of multiple RDEs which might be otherwise unaffordable with commercially available products. (As part of such a setup, there are multiple open hardware implementations of potentiostats/galvanostats in literature [8], [9], [10], [11])

**Fig. 1:**
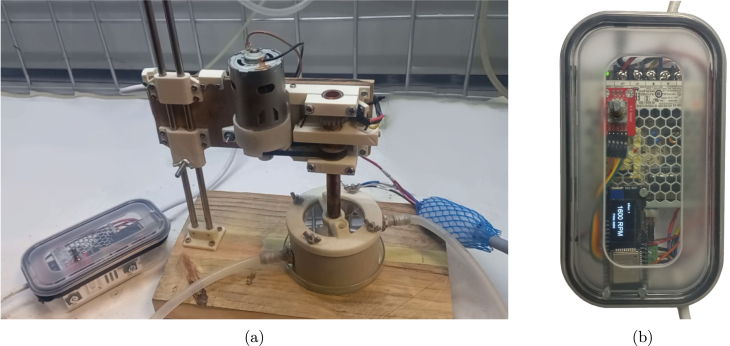
(a) The assembled rotating disc electrode prototype and (b) controller, using an ESP32 based microcontroller (Version HX2).

### 2.1 Mechanics and hardware

*Rotor and electrode mounting*

The rotor is assembled using a standard 15 mm copper plumbing pipe as the base. Copper was chosen for the prototype due to wide availability of both the pipe for the rotor shaft, and the closely sized straight copper pipe coupler to serve as a slip ring. The use of tube over a solid bar allows the accommodation of a 0.25 mm insulated wire (30 AWG, rated for 400 mA). Stainless steel tube is a suggested as alternative due to its greater corrosion resistance, with the caveat that it is often supplied in 6 m lengths. This would present the additional consideration of sourcing a suitable slip ring; should electrical isolation from the rotor shaft be desired.

The rotor shaft was electrically isolated from the current path to the electrode insert. This path included a copper slip ring cut from a 15 mm (nominal) straight copper pipe coupler, using PTFE tape as electrical insulation from the main shaft. Spring loaded carbon brushes contact the slip ring as the connection to the moving rotor. At the end of the rotor a sprung test probe to contact the removable electrode inserts for a reliable contact. Insulated wire (0.25 mm) is soldered from the slip ring to the test probe for a simple and direct connection between these parts. The test probe was supported in a PETG holder (*cap-base*) which has a screw thread to accommodate a *cap* into which the electrode insert is fitted, Fig. 3b.

Both, small carbon brushes from a Dremel rotary tool (Bosch Power Tools, Germany) and larger brushes designed for a *Baron* hanging motor (dentist/jewellers drill motor) were considered. Only the results of the latter are presented. The brush material specifications were not available from the local suppliers. Dremel brushes (4.8 × 5.4 mm) resulted in a high resistance after extended use while the larger carbon brushes (6.4 × 5.8 mm) provided stable performance. This is in part attributed to the larger brushes covering a larger arc of the collector ring making them less responsive to local height variations in the copper slip ring surface, which is not a precision machined part in this design. The hardness of brushes, friction coefficient between the brushes and slip ring and pressure on the spring will have an effect on the suitability of carbon brushes.

*Motor and gearing*

The DC motor used was rated for 7630 rpm at 24 V. The motor drives the rotor using a 6 mm GT2 timing belt and pulley, with a ratio of 1:2 ratio from the driving to driven pulley. The pulleys were 3D printed in PETG with M3 grub screws to lock them onto the shafts.

The use of a belt and pulley system is more tolerant to imperfect alignment between the motor and rotor compared to direct coaxial placement as is standard in commercial designs. This provides the additional advantage of electrically isolating the motor and rotor, and reduces direct vibration transfer.

A printed driving pulley was chosen over a commercially available aluminium equivalent. The commercial equivalent had a bore of 6.45 mm compared to the 6.35 mm shaft with 2 grub screws separated by 90°. This results in a small offset with the potential to affect the tension on the timing belt. In contrast the printed pulley was adjusted to a press fit with M3 grub screws symmetrically placed. PETG experiences less shrinkage and warping during printing, in part due to the lower required extruder and bed temperatures, making precise dimensions more easily achievable [12], [13], [14].

A pulley ratio of 2:1 (driven:driving) was chosen due to the speed capability of the driving motor and the size difference between the motor and rotor shaft diameters (8 and 15 mm respectively). The stall limit of the specified motor is relatively low near 240 rpm. With the 2:1 pulley ratio the rotor can operate at low speeds of a few 100 rpm while keeping clear of the motors stall limit. This ratio is suitable for the RDE’s intended operational range of 300–2000 rpm. It will most frequently be used at 1600 rpm, which is a common speed for the testing of catalysts for water electrolysis [15].

If higher speeds are required the driving pulley can be replaced and other factors, including increased vibration, and the potential for turbulent flow of the electrolyte become more significant concerns.

*Rotor and motor mount*

This serves to support rotor and supporting components. Included in this are the motor, tachometer, and brushes as per the design in Fig. 5, Fig. 3a. The *rotor mount* and *motor mount* serve as the central 3D printed parts. These are attached to an 8 mm thick supporting wooden board that provides rigidity and mounting point for attachment to a retort stand or custom mounting stand.

The *rotor mount* is printed in ABS (acrylonitrile butadiene styrene). The machinability and workability of ABS allows for easy sanding of the carbon brush slots to remove print defects and prepare the slots to allow the brushes to slide freely. Two version of this are provided, with and without the mount extending to provide an thread for a *motor mount* tensioner screw. In the version without, the smaller footprint was chosen to reduce warping of the printed ABS and the tensioner screw was mounted directly in the supporting board. PETG was used for the *motor mount* as is less sensitive to shrinking and warping as previously mentioned, facilitating the relatively large open shape of the motor cavity [12], [13], [14]. The *motor mount* is connected to the *rotor mount* with two M4 screws such that it can pivot to adjust tension in the timing belt connecting the motor and rotor which is controlled by an additional M4 screw.

*Electronics enclosure*

Depending on the operational environment, a 3D printed enclosure may be suitable. In the case of ABS, exposure to acetone is problematic as it is a solvent for ABS, while for PETG becomes brittle on exposure to KOH. As such a container made of these would require to be elevated off a surface using a chemically resistant material. As a simpler option a polypropylene container, reminiscent of a lunch box was selected to contain the electronics due to its chemical resistance. The enclosure was chosen to allow clearance above the power supply and a few centimetres space behind the MOSFET and linear regulator. The electronics PCB was spaced from the PSU with 3D printed *electronics clips*, Fig. 8(c). Components were fastened to this from the top and sides to not perforate the water-tight base. When choosing a container the chemical resistance and intended chemical environment need to be considered.

### 2.2 Electronics and power delivery

The electronics are built around a WEMOS LOLIN32, ESP32 based microcontroller with an on board SSD1306 128 × 64 px OLED display. This is based on, and can be substituted with the ESP32 microcontroller from *Espressif Systems*, China in combination with any 128 × 64 I2C display.

*Power delivery*

To power the motor, the microcontroller drives an n-channel MOSFET with a low trigger voltage using PWM signal at 9 kHz. This controls the current supplied to the motor from a *Mean Well* 12 V, 4.2 A power supply (PSU). The PSU was selected such that it is rated for close to twice the tested power draw of the motor. A PSU can be built/designed as an alternative to a commercial PSU. It is recommended that the PSU should offer safety features such as short-circuit, over-voltage and overload protections, regardless of whether a self-built or a commercial unit is utilised. Switching frequencies of 20 kHz are often used for similar applications to prevent an audible hum from the motor [7], [16], [17]. The lower switching frequency was selected to reduce switching losses which are dissipated as heat on the MOSFET. This allows operation without requiring a heatsink on the MOSFET. While there was a some operational noise from the prototype, there did not appear to be a significant contribution due to the choice of switching frequency for the chosen motor.

The microcontroller is powered using a LM7805 linear regulator.

*Tachometer*

The tachometer is based around the use of an optical gap sensor and a 3D printed PETG *tachometer wheel*. This wheel is mounted on the rotor shaft and has 4 evenly spaced fins. These fins block the beam of the optical gap sensor as they pass the sensor during rotation. The intervals between reading of the sensor are used to calculate rotational speed. The tachometer wheel is covered with aluminium tape for better defined signals on rotation; this is essential for natural PETG where the low optical density delays the response of the optical gap sensor. If available a dark or black pigmented PETG may result in the aluminium tape becoming redundant.

*User interface*

Control of the rotor operation and speed are provided through a rotary encoder connected to the microcontroller. Feedback is provided to the user on the microcontroller’s on-board OLED display.

**Fig. 2:**
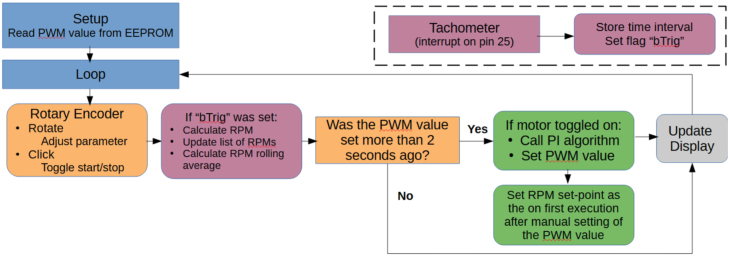
A flow diagram illustrating the software control loop. The PI algorithm and display calls are rate limited to 100 and 300 μ s intervals respectively.

### 2.3 Software

The electronics are controlled using the Arduino programming language, on an ESP32 microcontroller as part of the WEMOS LOLIN32. An earlier prototype made of use of MicroPython on a Raspberry Pi Pico with Nokia 5110 displays. The balance of the system was identical. Some of the data presented in Section 7 makes use of the earlier prototype. The factors affecting this redesign are detailed under “*Tachometer reading stability*” in Section 7, with additional data in SI Section 7.

*Tachometer*

The rotation speed is determined using a rolling average of the 8 most recent time intervals, measured during rotation between successive tachometer wheel fins. These are measured using an interrupt service routine (ISR) attached to the signal from the optical gap sensor.

The 8 interval rolling average used corresponds to 2 full rotations. Considering the tachometer wheel is a 3D printed rather than a machined part, the tolerances between the fins are not as precise as a machined part. If we were to distinguish the successive gaps between fins, by using a multiple of the number of fins, each new interval is replaced by a new value from the same gap for the rolling average. This prevents swings in the average due to the dimensional tolerances of the print.

The ISR additionally checks if the tachometer signal is *High* to bypass a well documented issue that prevents the ISR from differentiating a rising edge from a falling edge [18], it is unclear if there are exceptions to this behaviour.

The tachometer uses software based “debouncing”. The time elapsed between two successive accepted ISR call are compared against a minimum value. This is calculated from variables corresponding to a hard-coded RPM maximum and the number of fins on the tachometer wheel; represented by the variables *maxRPM* and *fins* respectively.

For an accepted ISR call, only the time interval and a boolean is set in the interrupt to minimise the computation time of the ISR. The remainder of processing and memory access processes occur in the main loop of the software, triggered by the boolean flag.

*Speed control*

The motor is controlled by a 9 kHz PWM signal using the ESP32 based microcontroller, this allowed for a PWM resolution of 13-bit corresponding to $2^{13}=8192$ discrete output values [19]. The PWM resolution was chosen to allow a fine enough adjustment for the PI control loop to operate on an effectively continuous adjustment in the context of a rpm unit.

The speed control uses the Arduino-PID-Library developed by Brett Beauregard [20]. A primarily I control loop is used with a small P contribution, with P, I, and D components of 0.5, 2, and 0 respectively and the output computed every 100 ms. The motor has a relatively low inertia responding quickly to changes in the output parameter preventing the sensitivity to windup in the I focused control. The P contribution assists in quickening the response to errors in the RPM setpoint. These values were optimised to the point of a suitable response, noting there is potential for a more aggressive parameter-set to control the given prototype.

*Operation logic*

The operation logic follows the software control loop in Fig. 2. The push button in the rotary controller is used to toggle the power delivery to the motor, setting the (*on/off*) state. The software control loop will set a PWM duty cycle for the MOSFET corresponding to motor speed which can be adjusted by turning the rotary encoder.

After a 2 s delay following user input, a PI control loop with a 100 ms interval will optimise the motor speed in RPM to the nearest 100 rpm (i.e. 1642 rpm will result in a 1600 rpm set-point). The delay allows the motor to accelerate/decelerate in accordance with the change in the PWM value. Operation will continue until the user presses the push button on the rotary encoder.

On change of state between *on* and *off*, the PWM value is written to persistent storage (EEPROM) if the RPM reading is within 5 RPM of the targeted setpoint. This value is read when the electronics are initially powered on for user convenience.

The PWM value is used for speed adjustment rather than setting an RPM set-point directly, due to the behaviour of an earlier prototype using MicroPython as the programming language. That version was subject to larger uncertainties in the tachometer readings. This was used in combination with a very slow PI control to prevent outliers from causing spikes in operational speeds, which made the PWM value the preferred means of speed adjustment. In contrast, the presented version offers a responsive PWM control.

**Design files**

## 3 Design files summary

**Table:** 

Design filename	File type	Open source license	Location of the file

Shnier 2024-RDE-SI.pdf	PDF	SHL-2.1	Available with the article
Clip_and_height_adjustment.mp4	Video	MIT	https://doi.org/10.17632/r4mh958pgm
Operation_and_adjustment_(20240724).mp4	Video	MIT	https://doi.org/10.17632/r4mh958pgm
RDE_Main_r0.6.8_GJbrushes_wider.FCStd	FreeCAD	SHL-2.1	https://doi.org/10.17632/r4mh958pgm
RDE_Main_r0.7.0_shorter-length.FCStd	FreeCAD	SHL-2.1	https://doi.org/10.17632/r4mh958pgm
RDE_GT2_driven_pulley.FCStd	FreeCAD	SHL-2.1	https://doi.org/10.17632/r4mh958pgm
RDE_motor_GT2_driving_pulley_20tooth_6p65_hole.FCStd	FreeCAD	SHL-2.1	https://doi.org/10.17632/r4mh958pgm
RDE_tachometer_wheel (three variants)	FreeCAD	SHL-2.1	https://doi.org/10.17632/r4mh958pgm
RDE_cap_base (two variants)	FreeCAD	SHL-2.1	https://doi.org/10.17632/r4mh958pgm
RDE_cap_r0.4_16mm-dia_14mm_5p2bore_025clear.FCStd	FreeCAD	SHL-2.1	https://doi.org/10.17632/r4mh958pgm
Electronics_clip (two variants)	FreeCAD	SHL-2.1	https://doi.org/10.17632/r4mh958pgm
M3_nut.FCStd	FreeCAD	SHL-2.1	https://doi.org/10.17632/r4mh958pgm
(Folder) RDE stand (FreeCAD and STL)	FreeCAD	SHL-2.1	https://doi.org/10.17632/r4mh958pgm
(Folder) Electrode insert mounting set	FreeCAD	SHL-2.1	https://doi.org/10.17632/r4mh958pgm
STL files for 3D printing (see description)	STL	SHL-2.1	https://doi.org/10.17632/r4mh958pgm
RDE_code.ino (v0.1)	Arduino Sketch	MIT	https://github.com/AShnier/RDE/releases https://doi.org/10.17632/r4mh958pgm

			

The FreeCAD [21] files in the above table are provided alongside STL files for 3D printing in the Mendeley data repository link provided.

*Design file descriptions*

**Fig. 3:**
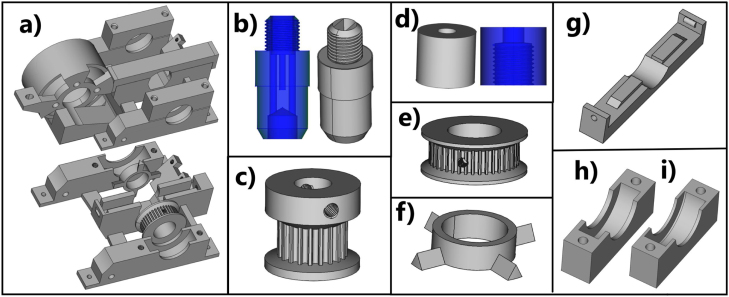
CAD models of the 3D printed parts used in the rotating disc electrode prototype. (a) Parts and positioning including the *rotor mount* and *motor mount* in Version HX1, (b) *cap-base*, (c) GT2 driving pulley, (d) *cap*, (e) GT2 driven pulley, (f) tachometer wheel, (g) brush holder clip, (h) *bearing holder bottom*, (i) *bearing holder top*.

**Fig. 4:**
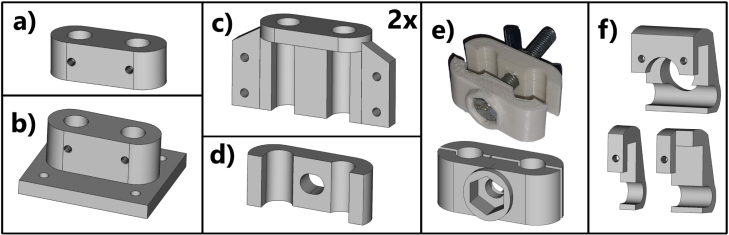
CAD models of the 3D printed parts used for the RDE stand. (a) *stand-top*, (b) *stand-bottom*, (c) *stand-attach*, (d) *stand-hold*, (e) *stand-stop*, (f) the variants of the *cable-clip*.

**Fig. 5:**
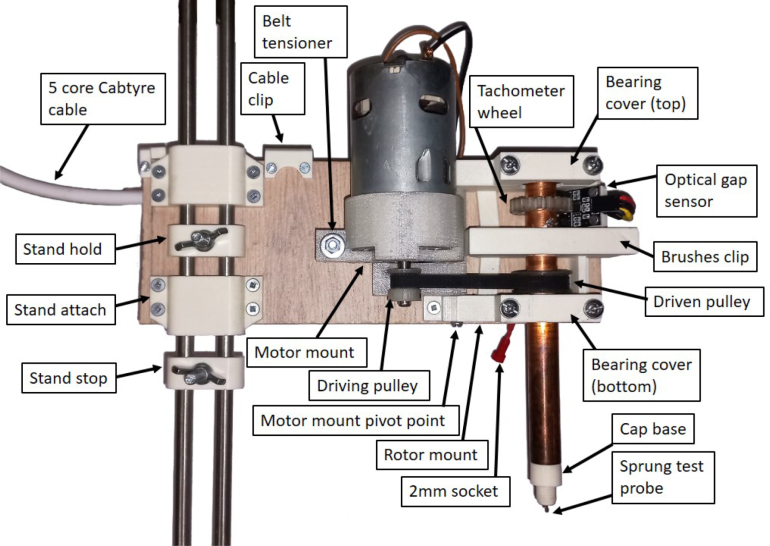
Labelled rotor assembly, Version HX2.

The full list of design file descriptions is provided in SI Section 11. Some files are provided with multiple variants, for these the end of the name in the data repository indicates the difference between the files. The file name directly corresponds to the specified parts in Fig. 3, Fig. 4, Fig. 5, With the exception of the *RDE_Main...* files described below.

• *Shnier 2024-RDE-SI.pdf* includes additional detail and supporting information on the fabrication, validation and design of the presented prototype.
• *RDE_Main_r0.6.8_GJbrushes_wider.FCStd*, The FreeCAD design file containing the *motor mount*, *rotor mount*, *bearing cover top*, *bearing cover bottom*, *brush clip*. This variant was used for collection of the results in Section 11.
• *RDE_Main_r0.7.0_shorter-length.FCStd*, The FreeCAD design file containing the same components as the above entry. This variant is more compact then the variant marked “wider”.
• *RDE_cap_base (two variants)*, The FreeCAD design file for the *cap base*, specifically *RDE_cap_base_r0.4.4 _PETG.FCStd* and *RDE_cap_base_r0.5.0_ABS.FCStd*. This part is press fitted into the end of the 15 mm copper tube. It holds the sprung test probe and the *cap* into which the sample inserts are fitted. The *r0.4.4_PETG* version was used for the results in Section 7.
• *RDE_cap_r0.4_16 mm-dia_14 mm_5p2bore_025clear*, The FreeCAD design file for the *cap* which holds a 5 mm diameter, 4 mm height insert to serve as a working electrode.
• *STL files for 3D printing*, these files are available in the Mendeley data repository. They have not been individually listed to avoid duplication of the FreeCAD files from which they were exported.
• *RDE_code.ino (v0.1)*, the Arduino code for the ESP32 based microcontroller. This is available on both GitHub and the Mendeley data repository.

**Bill of materials**

## 4 Bill of materials summary

The material comprising the prototype are detailed in Table 1

**Table 1:** Bill of materials.

Description	Qty	Unit	Total	Product link	Material type
		(USD)	(USD)		
**Motor and motor power**					
Motor 24V DC 5.6A 7630rpm	1	$14.90	$14.90	Mantech.co.za, (archived)	Other
Mean Well Power Supply 12V (10.2–13.8V) 50 W	1	$22.89	$22.89	Mantech.co.za, (archived)	Other
Schottky Diode 5A 60V	1	$0.46	$0.46	Mantech.co.za, (archived)	Semiconductor
N-Channel MOSFET IRLB8743PBF, Threshold <2.35 V	1	$1.75	$1.75	Mantech.co.za, (archived)	Semiconductor
Resistor 1K Ohm 1/4 W	1	$0.01	$0.01	Mantech.co.za, (archived)	Other
Resistor 10K Ohm 1/4 W	1	$0.01	$0.01	Mantech.co.za, (archived)	Other
3 Way Terminal Block	1	$0.34	$0.34	Mantech.co.za, (archived)	Composite
Rocker switch (rect.)	1	$0.38	$0.38	PiShop.co.za, (archived)	Composite
**Microcontroller, user input, and tachometer**					
Wemos Lolin ESP32 with OLED	1	$9.39	$9.39	PiShop.co.za, (archived)	Semiconductor
Optical Gap/Slot Sensor Board G=10 mm	1	$2.02	$2.02	Mantech.co.za, (archived)	Semiconductor
Rotary Encoder KE0053	1	$2.65	$2.65	Mantech.co.za, (archived)	Semiconductor
L7805 Linear Regulator 5V 1.5A	1	$0.49	$0.49	Mantech.co.za, (archived)	Semiconductor
Ceramic capacitor 0.33 uF 50V for L7805	1	$0.04	$0.04	Mantech.co.za, (archived)	Other
Ceramic capacitor 0.1 uF 50V for L7805	1	$0.01	$0.01	Mantech.co.za, (archived)	Other
Jumper Cable Kit 120Pcs 10 cm	0.3	$3.86	$1.16	PiShop.co.za, (archived)	Metal
Veroboard 65 mm × 145 mm, 5 pack	0.2	$4.03	$0.81	PiShop.co.za, (archived)	Composite
Bolt M3 × 6	6	$0.02	$0.09	Mantech.co.za, (archived)	Metal
(Alternative) Nut M3	0	$0.02	$0.00	Mantech.co.za, (archived)	Metal
Wire Solid 1.6 mm Red, 250 mm	1	$0.77	$0.77	Mantech.co.za, (archived)	Metal
Wire Solid 1.6 mm Black, 250 mm	1	$0.75	$0.75	Mantech.co.za, (archived)	Metal
3 Core Cable 1 mm${}^{2}$, per metre	2	$0.86	$1.72	LiteGlo.co.za, (archived)	Metal
5 Core Cable 1mmˆ2, per meter, Cabtyre	3	$1.44	$1.44	hardware store	Metal
Heat Shrink Tube Kit-164 pcs	0.5	$4.15	$2.07	Communica.co.za, (archived)	polymer
Polypropylene container	1	$0.99	$0.99	homeware store	Polymer
**Rotor and electrode**					
Copper Pipe 1.0 m 15 mm, 0.5 mm wall, Class 0	0.5	$4.42	$2.21	hardware store, (archived)	Metal
15 mm Straight Copper Coupler	1	$0.19	$0.19	hardware store, (archived)	Metal
Deep groove ball bearing 12 × 32 × 9 mm , NSK	2	$3.52	$7.05	BMGWorld.net, (archived)	Metal
(Alternative) Pulley, GT2, 20 Teeth for 6mrn Belt, 6.35 mm	0	$2.21	$0.00	3dprintingstore.co.za, (archived)	Metal
Closed Loop Timing Belt, GT2, 6 mm Wide, 200 mm	2	$1.10	$2.21	3dprintingstore.co.za(archived)	Polymer
Carbon Brushes (Baron Heavy Duty Hanging Motor)	1	$3.56	$3.56	gjsupplies.co.za, (archived)	Other
Spring Loaded Probe/1.5 mm Crown Contact 3A L =2 4.	5	$0.35	$1.75	Mantech.co.za, (archived)	Metal
Socket banana 2 mm	1	$0.97	$0.97	Mantech.co.za, (archived)	Composite
Silver plated copper wire, 30 AWG , max 400 mA, 300V	0.01	$28.75	$0.35	RS-online.com, (archived)	Metal
Grub Screw M3 × 3.0	4	$0.04	$0.18	Mantech.co.za, (archived)	Metal
Screw M4 × 35 plated	6	$0.04	$0.25	Mantech.co.za, (archived)	Metal
Screw M4 × 50 plated (*motor mount*)	1	$0.05	$0.05	Mantech.co.za, (archived)	Metal
Nut M4	3	$0.02	$0.07	Mantech.co.za, (archived)	Metal
M4 washer flat zinc plated	4	$0.02	$0.06	Mantech.co.za, (archived)	Metal
2.85 mm FDM filament: ABS natural	0.2	$13.82	$2.76	FilX.co.za	Polymer
2.85 mm FDM filament: PETG clear	0.1	$16.31	$1.63	FilX.co.za	Polymer
**Stand**					
228 × 38 mm pine base (priced per meter)	0.3	$4.49	$1.35	hardware store/recycled	Other
Stainless steel round bar, Grade 316, 8 mm, 6 m length	0.2	$22.71	$4.54	MetalCentre.co.za	Metal
M8 T-nut	4.0	$0.04	$0.15	Gelmar.co.za, (archived)	Metal
Levelling foot M8	4.0	$0.25	$1.02	Gelmar.co.za, (archived)	Composite
Grub Screw M3 × 3.0 (*Stand top, Stand bottom*)	8	$0.04	$0.36	Mantech.co.za, (archived)	Metal
Bolt M3 × 6	11	$0.02	$0.06	Mantech.co.za, (archived)	Metal
Chipboard screws, 4 × 30 mm (*Stand bottom*)	4	$0.02	$0.83	Gelmar.co.za, (archived)	Metal
Chipboard screws, 3 × 12 mm (*Stand attach*, *Rotor mount*)	12	$0.01	$1.05	Gelmar.co.za, (archived)	Metal
M6 × 40 mm bolt and wingnut	2	<$1.00	$1.00	hardware store	Metal

**Total**			$98.32

## 5 Build instructions

*General safety and best practices*

• A selection of safely considerations relevant to operation are highlighted in Section 6 *Operational safety*.
• During 3D printing keep clear of the printers hot head when it is at temperature.
• The stepper motors on the 3D printer used does not have crash detection. Clear the print bed before use and do not place you hands or other objects in the print area during operation.
• When 3D printing ensure the space is well ventilated, especially when printing ABS.
• Soldering irons are a burn risk. Set up the solder task such that your hands do not need to be near the soldering iron tip.
• Ensure all power is disconnected while working on the electronics or wiring.

*3D Printing of components*

3D models are provided in the FreeCAD file format to allow adjustment of sizes and tolerances for part substitution and accommodation of print tolerances of specific 3D printers. The print setting are provided in SI Section 10.

The choice of polymer/filament for this design depends on factors including the solvent and chemical species present in a specific use case. It is essential to verify the chemical compatibility for a specific use case against the polymers used in printing the RDE parts [22], [23].

The primary use case for this RDE prototype within the research group in which is was developed is for alkaline water electrolysis. Both PETG and ABS have a degree of resistance towards 1 M NaOH [22]. In the case of an electrochemical cell using 3D printed ABS or PETG cells lids for 1 M KOH; Our experience showed that PETG became brittle loosing its structural integrity within weeks. In contrast, the ABS equivalent has shown no sign of degradation over more than a year of regular use. However, ABS is unsuitable for use with many common organic solvents which fall outside of the our specific use case [22], [23].

*RDE main body*

M.1 (Optional) Use the FreeCAD software to customise the design files as needed for the sourced parts, such as the of carbon brushes, bearings, optical gap sensor.
M.2 Using a 3D printer, print the STL files from the associated Mendeley data repository ( link) separately using ABS for the rotor mount and associated clips/covers and PETG for the tachometer wheel, motor mount, driven pulley and driving pulley. These parts are labelled in Fig. 3, Fig. 5.
  • ABS provides a smoother finish which is preferred for the rotor mount as this includes the bearing slots.
  • PETG experiences less warping for more accurate print dimensions. It is noted that there are mitigations to reduce warping in ABS, and this is additionally affecting by air temperature and flow in the print environment.
  • (Optional) The printed threads can be tight on first use. An M4 screw with a pointed top can be used to open the threads. An M4 tap was tested in one build and resulted in loose threads which were easily stripped.
M.3 Following the measurements of the diagram in SI Figure 2b, cut the supporting board to size, 239 × 85 mm (Version HX2).
M.4 Drill the holes marked in SI Figure 2b with the exception of the higher set for one of the *stand attach* parts.
  • This includes pre-drilling 2 mm holes for chipboard screws.
  • For Version HX2, a hole is marked and drilled to place an M4 bolt to tension the *motor mount*, SI Figure 2b. For Version HX1, SI Figure 1a there is a thread in the mount for a M4 screw to be used as a tensioner. The design without built in M4 thread allows has a smaller footprint to reduce warping during printing with ABS with a 3D printer without a full enclosure.
  • The stand related parts are assembles with under the *Stand* heading.
M.5 Fasten the *rotor mount* onto a board, using chipboard screws (3 × 12 mm).
  • The board can be wood, a suitable plastic, or composite provided it is sufficiently rigid to act as a support. Raw chipboard is not well suited as it is a moisture sensitive material that has the potential to flake, which could contaminate its surroundings.
  • The board material used in the prototype was a 8 mm Egger Pro laminated floor board.
M.6 The *motor mount* is attached to the *rotor mount* at the *motor mount pivot point*, Fig. 5 using M4 bolts. These should glide freely through the corresponding holes in the *motor mount* to form the pivot point.
M.7 An M4 bolt is used as a tensioner for the GT2 timing belt. This is a simple alternative to the inclusion of an idler pulley, Fig. 5.
M.8 Mount the motor into the *motor mount* while ensuring the screw holes in the motor align with the round holes in the *motor mount*. The fit was tight, as such only a single short M5 bolt and washer was used to secure the motor to the enclosure. Check to ensure the ventilation holes of the motor are not obstructed.
  • A longer screw can be cut shorter. A jewellers saw is effective since the fine and sharp teeth will often leave the thread in tact.

*Stand*

The stand is composed of a wooden base, two lengths of 8 mm stainless steel (SS) round bar, 3D printed parts, and four sets of M8 T-nuts and levelling feet. The 3D printed parts are shown in Fig. 4.

S.1 Cut the wooden base to size, 300 × 228 × 38 mm. A thinner base can be used if a block is added to support the SS bar.
S.2 Drill a 9 mm hole, ∼30 mm deep in each corner of the base 20 mm from the edges for the T-nut. Hammer in the T-nuts.
S.3 Cut the thread on the levelling feet to ∼30 mm using a jewellers saw, then fasten the levelling feet in the T-nuts.
  • If a different type of saw or grinder is used, place a nut on the thread prior to cutting then use the nut to open the damaged thread once cut.
  • This step can be avoided by drilling the holes for the T-nuts through the base, which will leave the top of the thread visible from the top.
S.4 Drill 8 mm holes 20 mm apart, ∼30 mm deep for the SS bars, as per Fig. 1 and fit the bars into these holes. The 20 mm distance between these needs to be accurate while the placement is less sensitive.
S.5 Print *stand bottom* in ABS and slide it over the bars. Fasten it into the base with four chipboard screws. Tighten it against the steel bars using four M3 grub screws.
S.6 Print *stand stop* in abs and clamp into onto the bars using an M6 bolt and wingnut.
S.7 Print two *stand attach* parts, fasten one onto the mounting board ∼5.5 mm from the edge opposite the *rotor mount*. This is done by pre-drilling holes with a 2 mm drill bit followed by fastening of 3 × 12 mm chipboard screws.
  • The parts were a tight, yet permissive fit as printed. They were worked up and down the steel bars to loosen the fit prior to attachment.
S.8 Slide the board onto the bars and clamp the second *stand attach* part in place to mark the holes before drilling and fastening.
  • This step is to achieve good alignment between the two *stand attachment* parts
S.9 Print *stand hold* in ABS. Fasten an M6 bolt through the 5.5 mm hole from the back of the board and tighten a nut onto this as it feeds through. Then fit the *stand hold* part onto this bolt and secure with a wingnut.
  • If the M6 bolt cannot willingly be fastened through the 5.5 mm hole of the material being used, an M6 tap can be used to open the hole or a 6 mm hole can be drilled. A thread locker or cyanoacrylate glue (superglue) can be used to keep the nut tight if needed.
S.10 Print *stand top* in ABS and slide it onto the bars. Tighten it against the steel bars using four M3 grub screws.

*Rotor assembly*

**Fig. 6:**
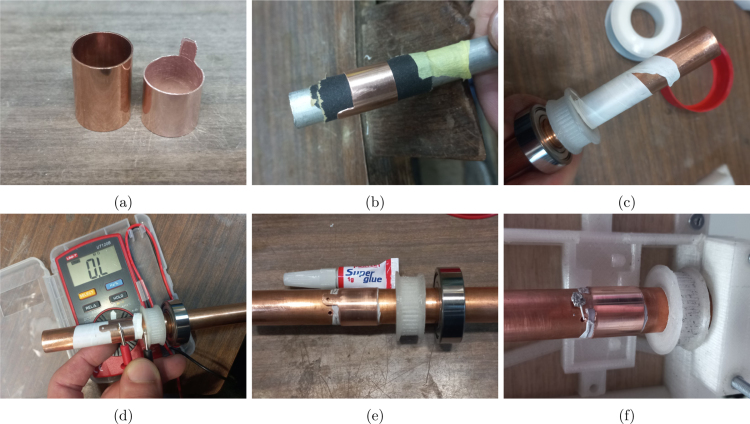
Slip ring preparation and assembly.

R.1 Print the *GT2 driven pulley* and *tachometer wheel* in PETG, the settings used are available in SI Section 10. Use a knife to clean and add a sub-millimetre chamfer to the bore of these parts.
R.2 The rotor is based on a 15 mm copper pipe. Cut the pipe to length with a pipe cutter leaving an additional 2 cm extra on both ends. This is to accommodate for imprecise placement of the bearing in the next step.
R.3 With appropriate PPE submerge the copper pipe in liquid nitrogen then quickly slide on the bearing which is closest to the middle of the shaft. If liquid nitrogen is not available the bearing can be heated with a hair dryer to a temperature slightly too hot to touch before pushing on the bearing quickly.
  • This is to take advantage of thermal contraction/expansion to allow easier fitting of the bearing.
  • Do not hit the bearing directly with a hammer. In the first version of this build, a hole was drilled in a section of 2 × 4” timber and this was used in combination with clamps to somehow press the bearing in place. A small precession was introduced in the process which is in part why a thermal expansion approach was used for the second version.
  • In addition to standard lab PPE, a face shield, cryogenic gloves and a cryogenic apron are required.
R.4 Once the bearing is on, mark out and cut the copper pipe to size using a pipe cutter or jewellers saw. Leave about 3 mm additional length past the end of the second bearing’s position.
R.5 Slide on the printed *GT2 driven pulley* and lock it in place with M3 grub screws. Its position is measured relative to the fitted bearing.
R.6 *Slip ring*, cut a 15 mm copper straight slip coupler as per Fig. 6(a) leaving a small tab for soldering on the 30 AWG insulated wire. Do not bend the tab yet as this will flatten the pivot point of the bend. Drill a 1 mm hole in the tab and deburr.
  • Copper straight couplers regularly have a indentation in the centre which acts as a stop when positioning two copper pipes for soldering. A slip coupler does not have this indentation.
  • The tab needs to be narrow to facilitate soldering as the wider it is the faster heat from a soldering iron will be diffused into the *slip ring*.
R.7 File a chamfer into both ends of the cut coupler, then work the diameter with 320 grit sandpaper held onto on a round stick or large drill bit end to increase the clearance with the 15 mm copper pipe. Finish with 600 grit sandpaper, Fig. 6(b).
R.8 Wrap 80 μ m PTFE tape with minimal overlap from the driven pulley well past where it will sit as a slip ring, Fig. 6(c).
  • Thinner PTFE tape has been effective on a build using larger overlap, the thicker 80 μ m tape is slightly less fragile during placement.
R.9 Slide on the slip ring on with a slight rotation in the direction of the PTFE wrapping. This is placed in a measured position centred with the brush holder with the tab pointing away from the fitted bearing.
  • Once placed, moving this is likely to break the PTFE insulation.
R.10 Once the slip ring is in place use a multimeter ensure there is no continuity between the shaft and slip ring as shown in Fig. 6(d).
  • If a resistance or continuity is measured, reapply a fresh piece of PTFE, restarting from Step R.8. It may additionally be prudent to check the slip ring for bumps or rough edges that could damage or catch the PTFE tape.
R.11 Cut some of excess PTFE leaving a few millimetres behind, recheck continuity and fix in place with cyanoacrylate (CA) glue, Fig. 6(e).
R.12 Once the CA glue is dry, drill a 1 mm hole slightly offset from where the slip ring tab is positioned and deburr the hole, Fig. 6(f).
R.13 Use a flat screwdriver or equivalent to raise the tab away from the main shaft. This is to allow soldering without contacting the shaft.
R.14 Cover *tachometer wheel* fins in aluminium tape. This was chosen as an alternative to ordering both clear and opaque PETG filament; due supplier specific limitations on the filament diameter. An opaque finish or opaque filament is expected to serve the same purpose.
R.15 Position the printed *tachometer wheel*, this should be a tight fit. If this is not the case the bore on the CAD file can be modified for a reprint. If its only slightly loose CA glue can be used to prevent movement.
R.16 Fit the second bearing as a push fit. It may be necessary to file a taper into the end of the shaft, and polish the shaft with 600 grit sandpaper.
  • For this part, it is an option to check the fit of the second bearing directly after fitting the first. This is with the caveat that after pushing the bearing partly on, it may be difficult to remove. For the Version HX1 build, the bearing could be removed and replaced by hand with some effort.
R.17 The rotor assembly is held in place with the **bearing cover top** and *bearing cover bottom* parts, Fig. 5. These are fastened in place with M4 bolts.
  • The top cover has more clearance in the direction of the rotor axis for a more forgiving placement of the second bearing.
R.18 Once the rotor is fitted in place, the slip ring should be polished using increasing grits of sandpaper (600, 1000, 2000) while rotating the shaft by hand.
  • The GT2 timing belt should be disconnected during this process.
  • The carbon brushes should not be in place for this process.
R.19 For regular maintenance, this process should be repeated every few months or earlier if unexpected noise is observed in the collected data.
  • The lowest grit of the previous step can usually be omitted for maintenance.

*Carbon brushes*

**Fig. 7:**
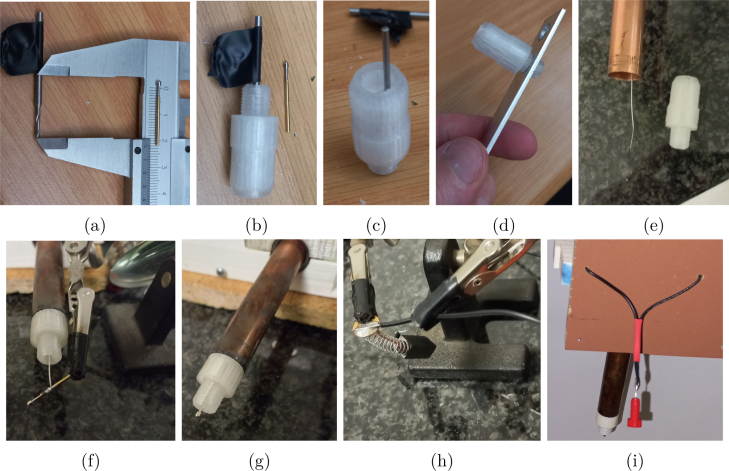
Cap base preparation (a, b) Tape was use used to mark depth on the drill bit, (c) aluminium bar with a tapped thread being worked in and out to clean the printed thread. (e, f, g) Inserting the *cap base* and soldering the test probe. (h) Soldering of brushes. (i) 2 mm socket prepared for soldering.

Br.1 Smooth the slots for the brushes using 320 or 400 grit sandpaper and finish with 600 grit. The brushes should be slightly too tight for the slots prior to sanding and the fit should be checked regularly until the brushes can slide freely in the slot.
  • When the brushes offered a tight yet permissive fit, the brushes placed would mark the high points with carbon indicating appropriate spots to continue sanding down.
Br.2 The carbon brushes fit into slots in the rotor mount which have notches to allow wires to pass from these slots.
Br.3 Set one of the carbon brushes into the *rotor mount* and note the position that the wire leaving the brushes slot needs to be orientated in. Solder of 1.6 mm OD wire onto the metal tab at the end of one of the carbon brushes, Fig. 7(h), The length should allow the wire to pass through the previously drilled hole and reach the bottom of the backing board Fig. 7(i) and SI Figure 2.
Br.4 Repeat for the second brush such that the placement of the wire is a mirror image of the first.
Br.5 Join and solder the two wires to the 2 mm socket such that the wires do not allow the socket to reach the rotor shaft. Heatshrink is recommended to insulate the solder joint, Fig. 7(i).
Br.6 Use the *brushes clip* to secure the brushes in their slot, one size has a clip and the opposite is secured with an M3 bolt.

*Cap base and sprung test probe*

B.1 Print the *cap base* in PETG or ABS using 0.1 mm layer height; a 30% fill was used for PETG and solid fill for ABS. Ensure a tight fit into the end of the rotor/copper pipe. A tight fit where glue is not needed allows the easy removal and replacement of this part, the FreeCAD model can be adjusted to ensure this fit.
  • This part can unintentionally be exposed to electrolyte. This can occur with the use of a worn out *cap* providing a loose fit for the inset, or if the electrolyte is pulled by the rotation up the shaft.
  • PETG becomes weaker and more brittle with exposure to 1 M KOH as observed in its use by the authors.
B.2 In aluminium or steel bar, drill and tap a hole corresponding to printed thread in the *cap base* and *insert holding caps*.
B.3 Open one of the through-holes with a standard 1.5 mm drill bit, Fig. 7(c).
B.4 Open the blind hole for the test probe with a 1.4 mm drill bit, this unusual size is to match the diameter of the test probe pins for a tight fit. Check the depth as you continue, and stop when the test probe if compressed will not protrude past the end of the *cap base* outer thread, Fig. 7(b).
  • The depth of the blind hole can be marked on the drill bit using tape, Fig. 7(a)
B.5 Feed an insulated 30 AWG wire through the hole next to the *slip ring*, down the length of the shaft, and feed it past the through hole in the *cap base*, Fig. 7(e).
B.6 Press-fit the *cap base* into the rotor.
  • This may require sanding down or cleaning the outside of the *cap base* with a blade to allow a gentle yet secure fit. If it seems like a struggle to press it, it is most probably too tight.
B.7 On the *cap base* side, strip the end of the wire and wrap it about 3 times around a sprung test probe slide out the test probe, slightly squash the winding such that it will be a contacting fit for the test probe.
B.8 Slide the winding halfway down the test probe. Support the test probe for soldering and solder quickly, Fig. 7(f). This is most easily done using a soldering clamp to hold the pin.
B.9 Solder the other end of the wire to the tab on the *slip ring*, Fig. 6(f). Allow some slack on the wire to sit inside the shaft. This make it easier to replace the sprung test probe if needed, as described in the SI Section 12.

*Insert holding caps*

C.1 Print the *cap* in natural ABS using 0.1 mm layer height and 30% fill with a raft.
  • The use of natural ABS is to bypass the consideration of whether, the pigments (which is some cases will be metal oxides) may leach into an electrolyte [24].
  • Fe and Cr based oxides are widely used pigments in plastics. These could be exceptionally problematic in performance characterisation of Ni based catalysts where Fe has the potential to cause a significant performance improvement [25], [26].
C.2 Use a bottoming tap matching the thread to open up and clean the printed thread. A M10x1 thread was used in the presented design to match existing equipment within our research group.
C.3 Lightly sand the external printed surfaces of the *cap* using 400 grit sandpaper.
C.4 Prepare an acetone solution saturated with natural ABS pieces. Hold the *cap* with tweezers or the bottoming tap and momentarily dip part of the *cap* into the solution, then allow the cap to dry.
  • The acetone smooths the *cap* surface which reduced drag in the electrolyte solution during rotation.
  • Dip symmetrically and do not submerge the *cap* past the tool that is being used to hold it. If there is pressure on parts of the *cap* that are wet with acetone, deformation is likely to occur.
C.5 Work a 5 mm drill bit through the hole for the inset by hand until the drill bit passes easily. *The 5 mm drill bit used was slightly smaller than 5 mm.*
C.6 Insert and remove an inset made from a hard material such a metal (See the insert removal tool SI Section 9. This serves as the final step of opening the hole.

*Electronics*

**Fig. 8:**
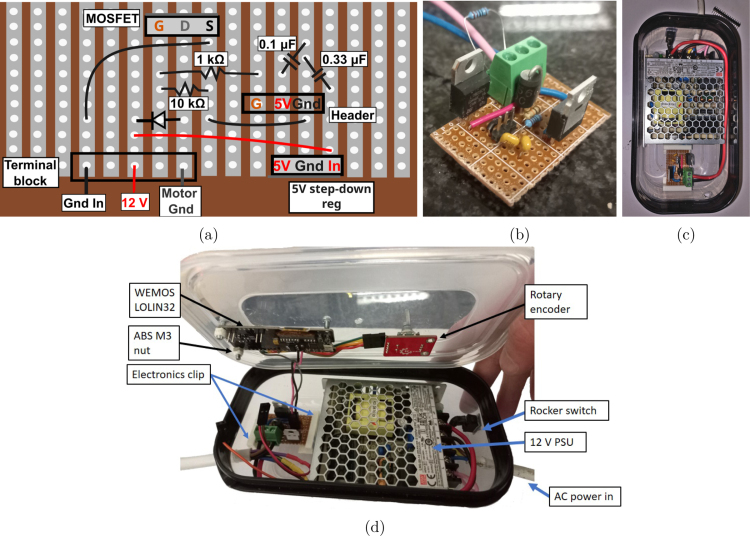
(a) PCB layout, (b) Cut PCB with a resistor as in the terminal block to allow testing of the electronics, (c) Open electronics box showing PCB in place with *electronics clips* before connecting the cable to the RDE, (d)

Soldering should be performed in a well ventilated area, ideally with a small fan or extraction unit to draw away the fumes. Soldering irons are a burn risk and fire hazard and should not be left unattended until they have cooled.

E.1 Solder the specified components onto the strip board as per Fig. 8(a) with a soldered board shown in Fig. 8(b).
E.2 Cut the strip board to size leaving at least 3 rows clearance on the short side and 1 row on the long size to allow for mounting, SI Figure 3a and Fig. 8(b).
  • A jewellers saw is ideal for this but a junior hacksaw sufficient.
  • Ensure the tooth direction is the saw presses the copper strips of the board towards the Bakelite. This is to prevent delamination of the strips.
E.3 Choose an appropriate enclosure, in our case we used a polypropylene enclosure for the chemical resistance of the material towards a wide range of aqueous bases, dilute aqueous acids, acetone, and alcohols.
E.4 Loosely place the PSU and electronics board to get perspective of the planned layout, verify and mark the placement of the holes for the incoming power cable, rocker switch, and cable or cables to the motor, Fig. 8(c).
  • Allow for a few centimetres from the back of both the MOSFET and linear regulator to any other components or enclosure wall to allow for cooling.
  • This prototype used a 5 core 0.75 mm^2^ Cabtyre cable to the motor with 2 cores to power the motor and 3 cores for the tachometer. This has a current rating of 6 A which is higher for the PSU used which is rated to deliver 4.2 A.
  • Any appropriately rated double-insulated cable should be suitable for carrying power to the motor. A separate set of wires can be used to connect the tachometer based on availability and preference.
E.5 Drill out a hole for the incoming power cable.
E.6 Cut hole for the power rocker switch.
E.7 Strip the power cable and cut the cores to length to connect live to the rocker switch, and neutral and earth to the PSU.
  • The power cable must not powered.
  • Use double insulated power cable.
E.8 Feed the power cable through the enclosure and feed the live wire back out through the rocker switch hole.
E.9 Slip heatshrink over the live wire, pass the copper through the centre hole in on the tab of the rocker switch and solder it in place to prevent movement.
E.10 Repeat the previous step for a brown or red insulated wire between the rocker and PSU.
E.11 Push the heatshrink over the tab to cover all exposed metal on the tab and shrink it with a heat source (heatgun, match or lighter).
  • Make sure there is nothing flammable and no solvents in the work environment.
E.12 Clip the rocker switch into the hole that it is hanging out of.
E.13 Fasten the PSU, using its mounting threads for M3 screws. Ensure the screws are not long enough to approach any internal components of the PSU.
E.14 Drill holes in the lid of the enclosure for the WEMOS LOLIN ESP32 with OLED display which can be fastened with three M3 screws and nuts. M3 nuts were printed in ABS to avoid conductive material from contacting any components on the board.
E.15 Drill a hole in the lid of the enclosure for the rotary encoder and its locking tab (the small metal piece which prevents rotation of the whole unit during use). Fasten this in place.
E.16 Connect the jumper cables as described in Table 2.
  • These connections can be made by direct soldering or using headers and jumper cables as desired.
  • The microcontroller specified included headers. These were cut into sections that were soldered to the utilised pins and PCB.
  • Cutting and soldering of the jumper cables can be used for connections where there is no movement as the thin wires become more brittle on soldering.
  • The connection to the rotary encoder was soldered on the microcontroller side using cut jumper cables.
  • The connection to the PCB was made with 1.6 mm (outer diameter) wires soldered on the microcontroller side. For the PCB, a header was used.
  • Thin 1.6 mm cable with a header connection were soldered into 5 core cable for the 3 tachometer connection points.

*Electronics adjustment*

**Table 2:** Connection guide.

Component	ESP32 Pin	Description
Rotary Encoder Gnd	GPIO 15	Ground
Rotary Encoder 3V3	GPIO 13	3.3 V
Rotary Encoder SW	GPIO 12	Click button
Rotary Encoder DT	GPIO 14	Rotary DT
Rotary Encoder CLK	GPIO 2	Rotary CLK
Tachometer Out	GPIO 25	Signal out
Tachometer 3V3	3V3	3.3 V
Tachometer Gnd	Gnd	Ground
PCB G^a^	GPIO 0	MOSFET Gate
PCB 5V^a^	5V	5 V supply
PCB Gnd^a^	Gnd	Ground

a The labels for the PCB are provided in Fig. 8(a).

A.1 Turn on the motor by pressing in the rotary encoder.
A.2 Adjust the PWM value using by rotating the rotary encoder towards a value 200 rpm above the maximum intended operating speed.
A.3 The PSU output voltage is adjustable. If the motor did not reach a high enough speed adjust the PSU output voltage using an insulated screwdriver.
A.4 If the motor reached the set speed, adjust down the supply voltage until the speed drops slightly below the setpoint.
  • By having the supply voltage no higher than necessary we minimise heating on the MOSFET and the linear regulator that supply the microcontroller. A lower voltage reduces the current passing though the MOSFET during switching. Additionally a lower voltage reduces the power dissipation for the linear regulator.
  • If heating is excessive on the MOSFET or linear regulator, then heatsinks should be added.

## 6 Operation instructions

*Operational safety*

• Be aware of the chemicals you are using and follow all the appropriate chemical safety protocols, including the use of protective gloves, goggles, lab coat, and other PPE as required.
• Ensure chemical compatibility between 3D printed parts of the RDE with the electrolyte and other chemicals in use. This is essential to avoid unwanted reactions with parts of the RDE.
• The rotor will throw off any liquids on rotation. If starting the motor under conditions where this can occur, such as outside of a electrochemical cell, any liquids should first be cleaned off rotor.
• The rotor rotates, as such do not wear anything that could get caught in the timing belt or anything that could be drawn in by the motor. This includes not wearing loose clothing, and tying up long hair.
• Keep the work space neat and clear. Items such as paper towel have the potential to get caught by the motor.
• Do not operate under explosive atmospheres or near flammable chemicals or materials. The motor used is not spark safe and is an open design to assist cooling.
• Do not adjust the RDE probe height while rotating. The RDE should not be touched while its rotating as it can be dangerous should anything get caught in the timing belt or other moving parts.
• Do not fully submerge the “cap” of the RDE in the electrolyte. This should not be submerged by more than half the height of the cap. This serves to prevent electrolyte ingress into the cavity behind the electrode via the joint between the *cap* and *cap base*.
• If the RDE is to be turned on while the *cap* is not covered/shielded by an electrochemical cell; the cap needs to be dried to avoid the spraying any residual electrolyte.
• The rotor shaft should not come into direct contact with the electrolyte. This can result in electrolyte contamination, unwanted reactions and/or corrosion of the shaft.

*Electrode insert mounting*

Once an electrode has been prepared use the insert mounting set to load the insert, SI Section 9. For printed ABS *caps* this is a water tight press fit. For machined PTFE *caps* Parafilm is wrapped around the circumference of the insert to form the seal.

*Reuse of insert mounting caps*

When using printed *caps* the porosity inherent to the printing needs to be considered. These should only be reused (after cleaning) for systems where contamination is not a risk factor. In a sample set where the electrolyte is identical, and the same set of elements are used, then reuse after cleaning is not expected to be problematic. The *caps* can be cleaned by sonication in deionised water (DI-H_2_O); compatibility should be considered prior to use with any other solvents. ABS printed *caps* remain water tight for a handful of runs. When the fit starts to become loose they need to be replaced.

*RDE using ESP32 microcontroller*

1. Check for obstructions around the rotor and timing belt.
2. Toggle on the on switch of the electronics enclosure.
3. (Optional) Adjust down the PWM value using the rotary encoder.
  • The PWM value is stored in the EEPROM when the rotor is turned off, this only occurs if the speed is within 5 RPM of the setpoint.
  • If the user does not know what rotation speed was last used, it is best to lower the PWM value prior to use.
4. Press the rotary encoder to start rotation.
5. Adjust the PWM value until the speed is comfortably within 50 RPM of the target value. The electronics will automatically define the setpoint as the nearest 100 RPM two seconds after adjustment. The delay is to allow the motor to reach the speed corresponding the PWM value.
6. Turn off the rotor by pressing the rotary encoder.
7. At this point we have checked the system is functioning as expected.
8. Turn off the power at the switch on the electronics enclosure.
  • The rotary encoder button only checks if the button is pressed. When this is the case, it repeats the check after 10 ms to prevent false reading from starting the motor.
  • While unexpected with the stated mitigation in place, a loose connection could cause unpredictable input. This could be dangerous should the motor be powered unexpectedly while changing samples. It is safer to turn off power to the rotor while loading and removing samples to entirely remove this risk factor.
9. Loosen the wingnut on the *stand hold* (Fig. 5), raise the RDE and tighten the wingnut.
10. Fit a *cap* with a sample/electrode inserted onto the rotor.
11. Set the height using the *stand stop* (Fig. 5), such that the *cap* is only partially submerged.
  • This allows the operation height to remain when changing samples and raising the RDE and keeping it up with the *stand hold*.
  • The *cap base* and copper shaft should never be submerged.
12. Check all the connections of your electrochemical cell.
13. Turn on the rotor as per Steps 2., 4., 5..
14. Collected data as needed.
15. Press the rotary encoder to turn off the rotor.
16. Turn off the power supply with the switch of the electronics enclosure.
17. Remove the *cap* and rinse the tip of the RDE with DI-H_2_O.

## 7 Validation and characterisation

This section demonstrates characteristics of the RDE and exemplifies its use for electrochemical experiments which depend on or benefit from the forced convection and associated mass transport effects. The quality of the results are discussed with the implications on the device and its use case.

*Tachometer reading stability*

The tachometer hardware is described briefly in Section 2.2. The development of this system was though modification of the tachometer wheel and software. The implementation initially used a MicroPython RP2040 microcontroller.

**Fig. 9:**
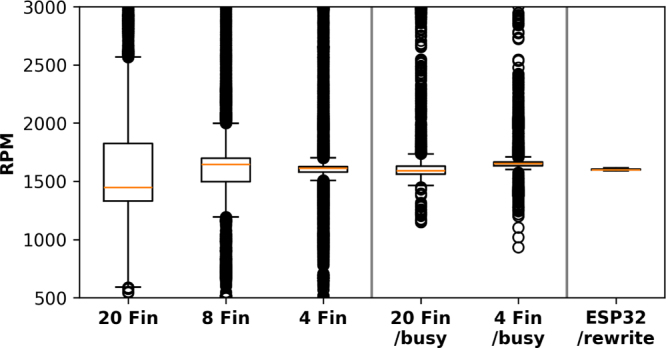
Tachometer sensor readings based on various designs with >20 000 raw readings per box plot, *busy* refers to a code variable *bBusy* which prevents the tachometer saving data during certain processor tasks.

The effect of the implementation variants is evident in Fig. 9. For the MicroPython control system, the motor speed was manually set using an external bench top power supply. The last 20 000 readings from data collection were used for the box plots. The ESP32 based system was controlled as per Fig. 2 used 36 745 readings after allowing 10 s for stabilisation, the stabilisation was enabled through the control loop correcting for drift in the motor speed.

The number of fins on the tachometer wheel is varied and where the label “busy” is used, tachometer reading are not taken during the more computationally involved processes including data processing and updating the display. We observed that using fewer fins, and limiting tachometer readings to exclude values from where there is any notable computation produced a narrower RPM distribution. The last box plot of this figure was collected using the C++ based code and ESP32 microcontroller produced the narrowest distribution.

The distributions for the RP2040/MicroPython implementations are primarily attributed to the behaviour of the default ISR method in MicroPython, where interrupts are scheduled resulting in a delay in execution by an unknown period on both sides of a time interval reading. Since this affects undifferentiated measurements on either side of an interval measurement, this can be mitigated as follows:

1. The reading can be statistically treated by selecting median values which are less likely to be distorted by large outliers.
2. The number of fins on the tachometer wheel can be reduced, which reduces the relative error by making the reading interval proportionally larger.
3. Pausing the reading of the tachometer during tasks which have the potential to result in longer wait times for the interrupts, such as processing of data or updating the display.

The combination of these approaches allows the effective use of a less than ideal RP2040/MicroPython implementation. This permits a control loop with a slow response to regulate speed using primarily integral control as presented and discussed in SI Section 7. It is noted that the authors are unaware of a means to implement more immediate execution of an ISR in this system using MicroPython. Alternative languages on the RP2040 and lower level coding, such as writing directly to registers was not explored for potential solutions [27].

To reduce the error in the measured data, a move was made to a WEMOS LOLIN32 board with a built in OLED. This was to address multiple problems of the previous implementation,

1. A PCD8544 was used in the initial design, this requires 8 wires. in contrast an on board display has all connections pre-wired simplifying the design.
2. The specific batch of three PCD8544 received had a poor connection between the panel and PCB on which it was supplied. This could be improved by placing a binder clip to put pressure where it makes connection, yet this was undesirable. An alternative was sought under the consideration “once bitten, twice shy”.
3. The immediacy of the ISR execution for the tachometer readings and moving to the faster C++ based Arduino language on the ESP32 based microcontroller.

**Fig. 10:**
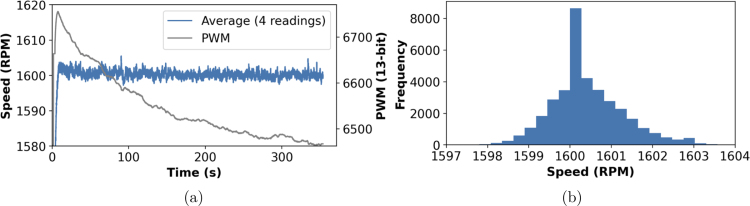
Tachometer readings using the ESP32 based implementation with a PI control loop of P = 0.5, I = 2 applied to a 13-bit resolution PWM signal. Values are presented as a 4 reading moving average. (a) The PWM values and speed vs time. (b) Distribution of RPM values from 10 s onwards.

This allowed for a responsive PI control loop as presented in Fig. 10(a) showing data from a set-point of 1600 rpm. This produced a narrow range of RPM values, as shown in Fig. 10(b) with a mean of 1600.41 rpm, median of 1600.25 rpm, and std. dev. of 0.91 rpm.

It is noted that the Arduino language can be used on a RP2040 [27]. Once the decision had been made to rewrite the code, it was considered preferable to move to the board with an onboard display during this process.

*Resistance stability*

In electrochemistry, the electrochemical response of a system is used to determine specific properties of this system. In doing this, both the electrochemical properties of the system and impedance properties of all components involved will affect the measurements. In the case of the resistance across the RDE, this follows Ohms’ law V=I⋅R where a potential drop is observed in proportion to the resistance. While the resistance value can be compensated for, the stability of the resistance value directly impacts the precision of the measurements. This stability is primarily affected by the brushes contact, as established in literature. This will in turn determine the noise level in the measurement of current [2].

Graphite brushes are reported to drift in resistance with time [28]. Many commercial RDEs make use of silver-graphite brushes and in this implementation electro-graphite is utilised. For these types of electrical contacts it is necessary to validate the stability and error in data collection associated with resistance drift for the RDE. For this we consider a series of electrochemical measurements during the oxygen evolution reaction in alkaline water electrolysis. The experimental details are provided in SI Section 6.

**Fig. 11:**
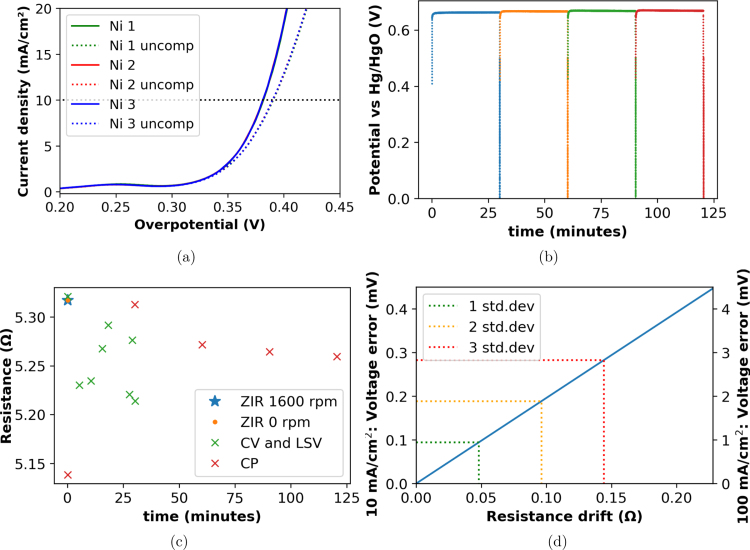
Characterisation of a Ni electrode in 1 M KOH using the prototype RDE in the context of the oxygen evolution reaction in alkaline water electrolysis (a) LSV at 10 mV s^−1^, (b) CP with internal resistance measurements at 0 V every 30 min, (c) Internal resistance (IR) values measured at 100 kHz between each technique, with the order of techniques LSV, CV, and lastly CP. (d) Voltage error vs resistance drift for 10 and 100 mA cm^−2^ conditions with values at multiples of the standard deviation from the values in (c) (5 mm electrode insert). (Collected using with rotation rate set using an adjustable bench-top power supply and manual speed calibration.)

A bare nickel electrode was prepared and conditioned using cyclic voltammetry. For the reaction considered, the over-potential (η) is a critical reaction performance criteria. This was determined for three linear scan voltammagrams (LSV) with 85% IR compensation in Fig. 11(a). The traces overlap well with over-potentials at 10 mA cm^−2^ ($\eta_{10}$) of 381.6, 381.3, and 381.2 mV for the 1st, 2nd and 3rd scans respectively. This is a tight agreement of the successive scans. Over a longer time period chrono-potentiometry (CP) was used as in indicator of consistency, Fig. 11(b). The CP data shows smooth data with a slight increase with time, this is compared to the IR values in Fig. 11(c). We observed a change between the first two IR values of 0.17 Ω followed by 3 values within a 0.02 Ω deviation. It needs to be considered that we have an active reaction affecting the electrode surface, where the potential values reached with the preceding LSV’s are expected to alter the surface species on the electrode. The magnitude of voltage error due to resistance drift is presented in Fig. 11(d). This does not distinguish effects due to the electrode state from those from the current path from the instrument to the electrode. For a 5 mm diameter electrode, an error within two standard deviations of the values in Fig. 11(c) will present an error of less than 0.2 mV when measuring $\eta_{10}$ and below 2 mV for higher currents at $\eta_{100}$. The steady state CP measurements are expected to have less variation in the resistance value due to the state of the electrode surface. If we consider this as more indicative of the resistance drift due to the RDE itself, the voltage error will be smaller by more than a factor of 5.

### 7.1 Application example

*Diffusion coefficient determination*

The diffusion coefficient (D) relates flux of a chemical species to a concentration gradient as described by Fick’s first law, where the flux is proportional to the Faradic current at the electrode surface [29]. This illustrates a basis for relating Faradaic current, the diffusion coefficient, and bulk solution concentration. In the case of a stationary electrode, the distance across which there is a concentration gradient, the diffusion layer has a time dependence. With a RDE, the time dependence is replaced by dependence on the angular velocity of the electrode. This is expressed in the Levich equation to relate diffusion limited current, $i_{d}$ (A cm^−2^) to bulk solution concentration, ${\left[B\right]}_{bulk}$ (mol cm^−3^) of a species using the diffusion coefficient, D and angular velocity, ω (rad s^−1^) of the electrode:

(1) $$i_{d}=0.62\cdot nF{\left[B\right]}_{bulk}D^{2/3}\nu^{-1/6}\omega^{1/2}$$

where, n: number of electrons transferred for the half reaction, F: Faraday constant (C mol^−1^), ν: Kinematic viscosity of 0.1 M KCl in H_2_O is 0.0110 cm^2^ s^−1^ at 16 °Celsius, this was extrapolated from the value for H_2_O using data from Kestin et al. [30], [31].

In combination with a RDE, this can be used to produce a Levich plot of the diffusion limited current vs. the square root of angular velocity for the determination of the diffusion coefficient. An RDE offers an advantage over static experiments since the measured current is at steady state [29]. The Levich equation assumes a coaxial alignment of the active surface of the electrode with the axis of rotation, deviations from this are expected to result in an increase in the measured current, and consequently a larger D [29]. Bard et al. finds this as unlikely to be problematic in practice for the low to moderate rotation rates that the prototype RDE is limited to [2]. Considering the design of the current prototype; where we are evaluating convenience and accessibility rather than more ideal components, such reassurance cannot be assumed. Additionally the stability and linearly of the Levich plot can be evaluated as a representation of the RDEs’ performance [29].

The Levich equation requires use within its validity range. It depends on a sufficiently small hydrodynamic boundary layer and laminar flow to the electrode. The hydrodynamic boundary layer becomes smaller with increasing rotation rate requiring a rotation rate, $\omega \left(s^{-1}\right)>10\frac{\nu }{r^{2}}$ (r: probe radius). At higher speeds the laminar flow is disrupted and turbulent flow starts to occur. The transition point is dependent on the Reynolds number, which is a function of the probe radius ($Re=\frac{\omega r^{2}}{\nu }$). For this specific definition of Re, as described by Bard and Falkner, deviations are expected from laminar flow above a critical Re value of $2\cdot 10^{5}$ [2], [29]. The transition to turbulent flow is reported to occur at lower values of Re, due to factors including surface roughness of the probe/electrode, deviations from ideal rotor shaft alignment, and/or electrochemical cells walls that are too close to the electrode surface [2], [29], [32]. Empirically a RDE is expected to exhibit turbulent flow at speeds roughly approaching 10 000 rpm [2], [29].

For the purpose of a Levich plot, an equimolar solution of 5 mM K_3_[Fe(CN)_6_] and K_4_[Fe(CN)_6_]⋅ 3H_2_O in 0.1 M KCl was prepared as per SI Section 5. Cyclic voltammetry (CV) at 10 mV s^−1^ were collected using a 5 mm diameter Pt working electrode (WE), Pt counter electrode and saturated calomel reference electrode. Prior to use, the Pt WE was electrochemically polished as described in SI Section 5.

**Fig. 12:**
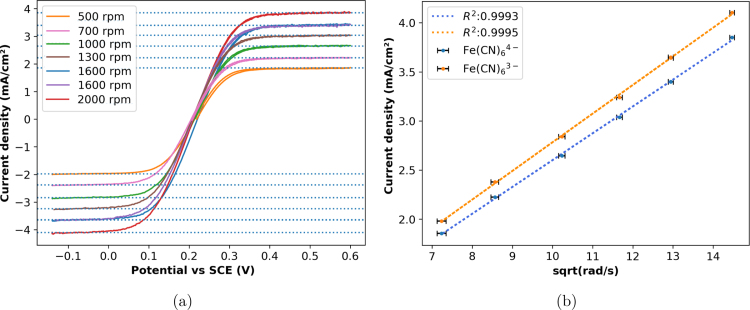
Electrochemical characterisation of an equimolar 5 mM K_3_[Fe(CN)_6_], K_4_[Fe(CN)_6_]⋅ 3H_2_O solution using RDE Version HX1 (a) CV with saturation current densities indicated by horizontal lines, (b) Levich plots of the absolute saturation current density for oxidation of Fe(CN)_6_^4−^ and reduction of Fe(CN)_6_^3−^ species.

The current plateaus from CV were used to determine mass transport limited current densities using the third cyclic voltammogram of each set at −0.08 and 0.55 V with limiting currents indicated by horizontal lines, Fig. 12(a). A slight hysteresis is observed and more pronounced at lower rotational speeds which is consistent with the data in Biologic’s EC-Lab Application note # 56, and both commercial and in-house produced RDEs in the work of Wittingham et al.; This is attributed to the 10 mV s^−1^ scan rate being sufficiently fast to prevent the system reaching equilibrium in this region [3], [7]. These current densities were used for the Levich plots in Fig. 12(b) where linear regressions were used to determine the gradient for use in Eq. (1). There is a temperature difference between the literature and collected data. This is adjusted for by using the appropriate kinematic viscosity. These values are 8.95e−06 and 9.88e−06 cm^2^ s^−1^ for Fe(CN)_6_^4-^ and Fe(CN)_6_^3-^ respectively ($R^{2}$ of 0.9993 and 0.9995 respectively). This work produced slightly higher values than literature, with literature values determined using a Levich plot and RDE providing the closest comparison, Table 3. The measured diffusion limited currents were highly sensitive to the cleaning process of the Pt WE, as shown in SI Figure 4 where a comparison between the use of mechanical vs electrochemical polishing as the final step is provided. Without the additional step of electrochemical polishing by CV in H_2_SO_4_, lower currents and a lower diffusion coefficient was determined, SI Section 8. This consistent with electrode poisoning effects due to storage under atmospheric conditions [1]. The diffusion coefficients determined in this work are slightly higher than literature values, with the deviation arguably in line with the uncertainty of the experimental details.

Beyond the values obtained, the collection of data for a Levich plot offers an example of a hydrodynamic experiment suitable for education purposes. If the Pt counter electrode is used as the voltage reference, the CV trace becomes centred at 0 V. The experiment can then be modified to replace the CV with chronoamperometry for a simplified, manual version of the data collection using a bench-top power supply with a built in or external ammeter.

**Table 3:** Literature diffusion coefficients of  and  species.

		Medium	Details	Ref	year
$10^{-6}$ cmˆ2 sˆ-1	$10^{-6}$ cmˆ2 sˆ-1				
6.5	7.7	0.1 M KCl		[33]	1953
6.6	7.6	0.05 M KCl		[33]	1953
7.35 ± 0.05	8.9 ± 0.1	H_2_O	calculated^a^, 298.15 K	[33]	1953
7.35	8.96	H_2_O	calculated^a^, 298.15 K	[34]	1993
8.0	8.9	0.5 M K_2_SO_4_	Levich plot	[35]	1972
8.95	9.88	0.1 M KCl	Levich plot, 289.15 K^b^	This work	2024

a Values calculated from ion conductivities and assumes infinite dilution.

b Electrochemically cleaned using CV in 1 M H_2_SO_4_.

### 7.2 Capabilities and limitations

• Suitable for the measurement of cylinder shaped electrode insets with or without surface coatings.
• Operating speed: 300–2000 rpm.
• Speed correction to the nearest 100 rpm based on tachometer readings.
• Simple controls and display for speed readout (Rotary encoder with a press switch).
• Easily repairable with inexpensive parts.
• Appreciable precision and measurement stability, with a RPM standard deviation of 0.91 at 1600 rpm.
• Not suitable for speeds significantly over 2000 rpm due to the low precision fabrication.

### 7.3 Conclusion

The production of the prototype allows application in low budget environments within and beyond South Africa, enhancing accessibility for research and education proposes. The high cost of commercial RDEs can be prohibitive in many environments. This is magnified in cases where multiple units are required to allow efficient or simultaneous work, such as were extended stability testing of systems is required, or in education where there is a limit to how many students can reasonably share an experimental setup. An example of an experiment suitable for education purposes based on the Levich equation and plot was discussed at the end of Section 7.1.

The prototype RDE presents reproducible performance with a known variation in the RPM values within ±3 RPM of the set-point as evident in the histogram showing the distribution where this data presented a standard deviation of 0.91 RPM, Fig. 10(b). The PI control loop facilitates correction for drift in the motors performance as will occur with temperature, run time, and age. There is room for further optimisation of the control loop parameters yet this will be motor specific.

The RPM control results in a linear trend as a function of rotation rate in the Levich plot indicative of good performance. The determined diffusion coefficients are slightly higher than literature with the source of the variation uncertain between the effect of variations between experimental conditions vs. an intrinsic effect of the prototype rotor.

This is prototype ideal for comparative investigative work, such as across samples measured with the same system and for stability testing where the comparative change in sample performance is of primary interest. It is suitable for experiments requiring up to the artificial speed limitation of 2000 rpm. Higher speeds were not explored as this would require a higher voltage PSU and a modification of the electronics to include heatsinks on the MOSFET and linear regulator powering the microcontroler. In the case of high precision work looking at the nth decimal place of measured values, the small variation in the RPM value, and the effect of any other properties which may result from the low precision production needs to be determined against a trusted commercial alternative.

This is a design of compromises, the copper rotor is sensitive to corrosion. A higher quality version can be made by moving to a stainless steel rotor, with the challenge of sourcing or machining a suitable slip ring and spacer for the diameters available. A design can be produced with direct contact between the rotor shaft and brushes. Here the larger size, which would include contact with the bearings, has the potential to be more susceptible to electromagnetic interference. The effect hereof has not been considered in the present work [36]. Additionally, the separation of the rotor from the motor by means of a timing belt allows for a less precise alignment between these parts.

A fully covered enclosure is suggested as a safety device and to reduce the possibility of contamination of an experiment from particulate; due to wearing of the brushes and/or timing belt. Substitution of a spark safe motor and moving the electronics completely out of the experimental area are required as the first steps towards working with flammable solvents or gases, with the electrical contact between the carbon brushes as an additional consideration.

Overall, the RDE prototype offers exceptional utility with the advantage of not requiring the use of precision machining tools in the build process adding to its accessibility.

## CRediT authorship contribution statement

**Adam Shnier:** Writing – review & editing, Writing – original draft, Software, Methodology, Investigation. **Tarisai Velempini:** Writing – review & editing, Investigation. **Anzel Falch:** Writing – review & editing, Supervision, Resources.

## Declaration of competing interest

The authors declare that they have no known competing financial interests or personal relationships that could have appeared to influence the work reported in this paper.

## Appendix A Supplementary data

MMC S1 The supplementary information contains additional figures, experimental information, data, maintenance information etc..
