# Supplementary material for: Low cost rotating disc electrode built using accessible hand tools and 3D printing
Source: HardwareX. 2025 Jan 31;21:e00626. doi: 10.1016/j.ohx.2025.e00626 (PMC11848141; doi:10.1016/j.ohx.2025.e00626)
Supplement: MMC S1 — The supplementary information contains additional figures, experimental information, data, maintenance information etc.. [file mmc1.pdf]

Supplementary information for: Low cost rotating disk electrode built using accessible hand tools and 3D printing

(Version 23 July 2024)

Contents

1 Terms and abbreviations 1

2 Image processing 1

3 RDE hardware versions 2

4 Additional figures 2

5 Experimental procedure for $K_3[Fe(CN)_6]$, $K_4[Fe(CN)_6] \cdot {}_3H_2O$ characterisation 4

6 Experimental procedure for OER with a bare Ni electrode 4

7 Comparison between RP2040 with MicroPython and ESP32 with Arduino code 5

8 Levich analysis without an electrochemical polishing step 5

9 Sample loading 6

10 3D Printer settings 6

11 Design file descriptions (full list) 7

12 Replacing a sprung test probe or cap base 8

13 Failed searches for 12V DC motors with a built in encoder or sensor 9

14 Three phase motor option 9

1. Terms and abbreviations

- **ABS,** acrylonitrile butadiene styrene; a thermoplastic polymer
- **CP,** chrono-potentiogram
- **CV,** cyclic voltammagram
- **EIS,** electrochemical impedance spectroscopy
- **IR,** internal resistance
- **LSV,** linear scan voltammagram
- **PETG,** p terephthalate glycol; a thermoplastic polymer
- **PPE,** personal protective equipment
- V_{OC}, open circuit potential

2. Image processing

Select images were processed for clarity in using brightness, contrast and shadows tools from *GNU Image Manipulation Program* (*GIMP*). Background removal was carried out using *Krita* for CAD model representations

and www.adobe.com/express/feature/image/remove-background for photos. *Krita* was used to blur parts of the background of some images without affecting the subject of the image. The addition of text boxes and arrows to figures was carried out in *Microsoft PowerPoint*.

Figure 8a showing a diagrammatic representation of the electronics layout on a PCB strip board was created using *Inkscape*.

3. RDE hardware versions

SI Figure 1a presents the HX1 RDE version in operation during electrochemical measurements. This makes use of a slightly simpler mounting mechanism where the supporting board was fastened directly to a laboratory retort stand using chipboard screws and plastic blocks that are designed as corner joiners, Modesty Blocks (archived). The central hole in these blocks was cut open to one side to allow the blocks to sit over the retort stand.

SI Figure 1b presented a rotor mount with a smaller footprint where the motor tensioner fastened using a M4 bolt in the backing board itself. This is a full height photo to show the *stand top* part.

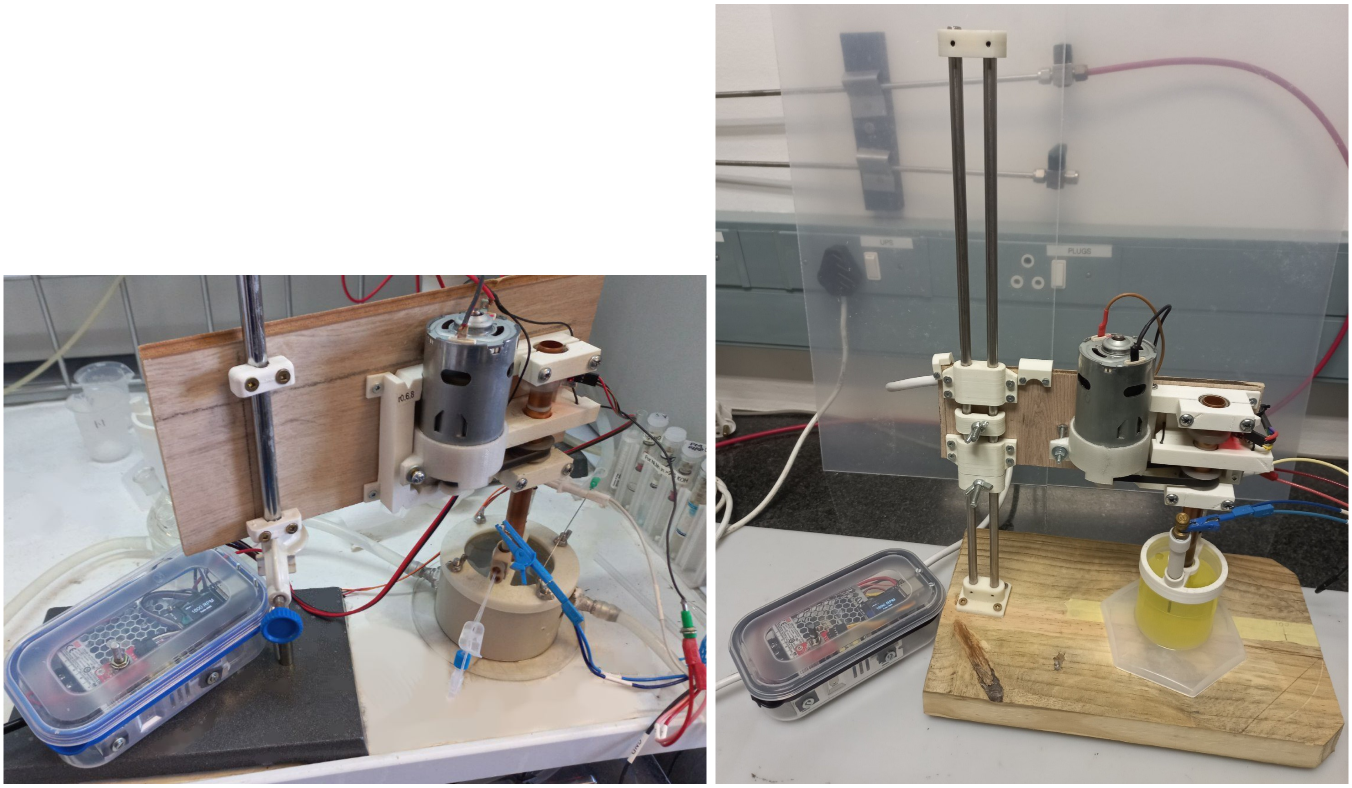

(a) (b)

Figure 1: (a) RDE version HX1, the assembled rotating disk electrode prototype and controller, using an ESP32 based microcontroller. with motor tensioner thread on *rotor mount*. (b) RDE version HX2 with stand.

4. Additional figures

Additional Figures are provided in SI Figure 2 and 3.

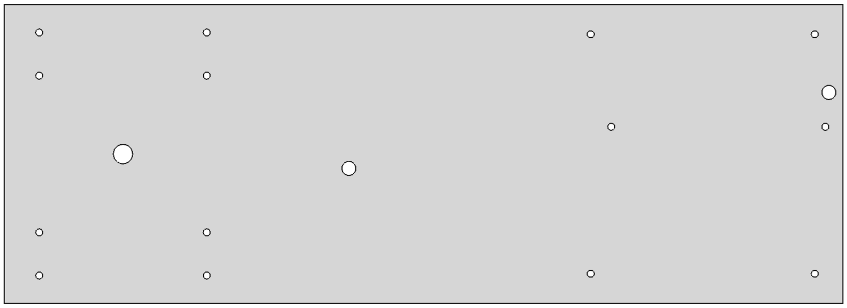

(a)

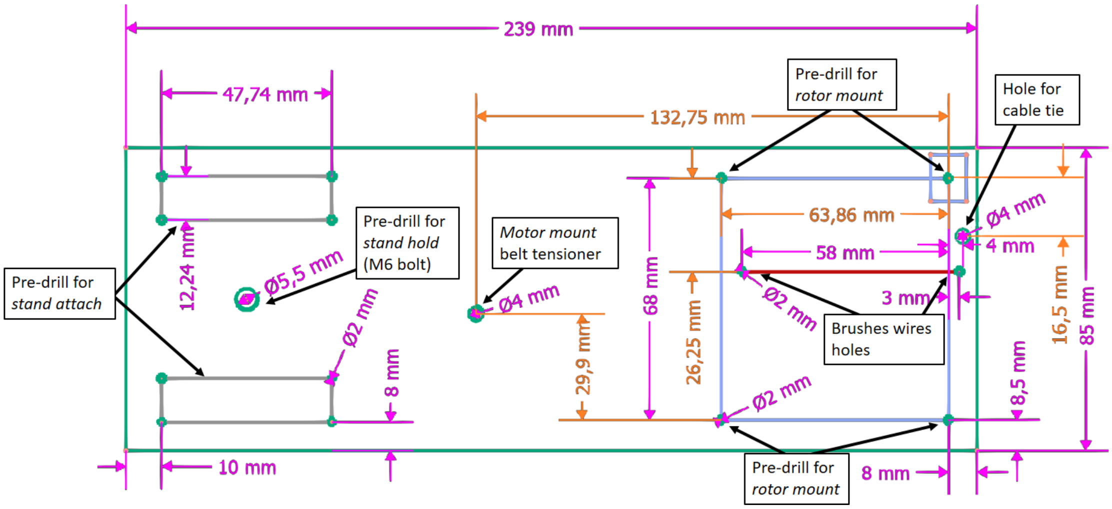

(b)

Figure 2: Holes drilled into the mounting board for Version HX2 (a) an unobstructed view, (b) labelled and measured.

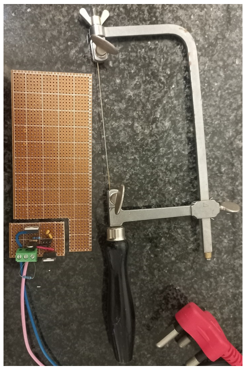

(a)

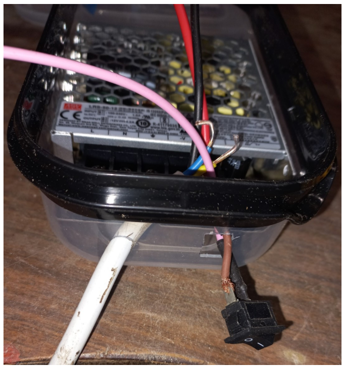

(b)

Figure 3: (a) Full and cut PCB, (b) Power switch prepared for soldering.

5. Experimental procedure for $K_3[Fe(CN)_6]$, $K_4[Fe(CN)_6] \cdot {}_3H_2O$ characterisation

Chemicals,

- $K_3[Fe(CN)_6]$ from Sigma-Aldrich 702587-50g,
- $K_4[Fe(CN)_6] \cdot {}_3H_2O$ from LABCHEM, CP grade,
- KCl from Sigma Aldrich 409316-25G,
- Millipore deionised H_2O

Procedure, A 250 (ml) solution of 5 mM $K_3[Fe(CN)_6]$ and $K_4[Fe(CN)_6] \cdot {}_3H_2O$ in 0.1 M KCl was prepared using Millipore deionised water. Powders were weighed accurately to within 0.1 (mg). This was then measured using a thee electrode cell maintained at 25 °C with Pt working (5 mm diameter), Pt counter, and SCE reference electrode. The cell was filled with 120 ml of this solution and purged with N_2 gas for no less than 30 minutes. Version HX1 of the RDE was used for these measurements.

The Pt electrode was mechanically polished using 1 μm diamond polishing solution followed by electrochemical polishing using cyclic voltammetry at a 250 mV s^{-1} scan rate in the range -0.3 to 1.3 V and -0.3 to 1.5 V with a set composed of 11 cycles of the former, 4 of the latter, followed by 3 of the former. This set was repeated until well defined, reproducible features were observed in the hydrogen region of the measurement. The effect of the electrochemical polishing on the measured limiting current is evident in SI Figure 4.

Three cyclic voltammograms were measured at 10 mV s^{-1}. N_2 gas was used to blanket the cell during measurements, the cell was additionally purged for no less than 2 minutes between rotation speeds.

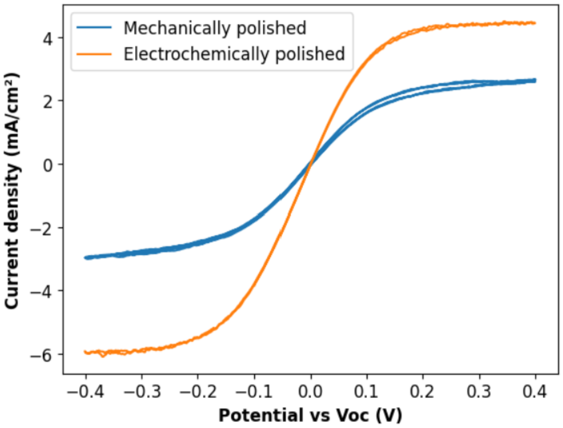

Figure 4: A comparison between a mechanically vs electrochemically polished Pt electrode using cyclic voltammograms of 5 mM $K_3[Fe(CN)_6]/K_4[Fe(CN)_6]$ system measure with rotation at 1600 rpm.

6. Experimental procedure for OER with a bare Ni electrode

Chemicals,

- KOH from Sigma-Aldrich 221473-1KG, > 85 % purity
- Millipore deionised H_2O

Procedure, A 1 (l) electrolyte solution of 1 M KOH was prepared using Millipore deionised water. This was then measured using a three electrode cell with bare nickel working (5 mm diameter), glassy carbon counter, and Hg/HgO reference electrode. Measurements were conducted at a controlled room temperature of 19 °C. The Hg/HgO electrode was calibrated against a saturated calomel electrode. The cell was filled with 130 ml of this solution and saturated with O_2 gas for no less than 30 minutes.

The series of measurements was collected in the order of electrode conditioning with CV at 100 mV s^{-1}, CV at 10 mV s^{-1}, three LSVs, and four 30 min CPs. Between each measurement an IR measurement was taken at 100 KHz. For the LSV measurements, IR was corrected for at 85 % using the average of the IR measurements collected directly before and after each LSV. The IR values are presented in Figure 11c of the manuscript.

7. Comparison between RP2040 with MicroPython and ESP32 with Arduino code

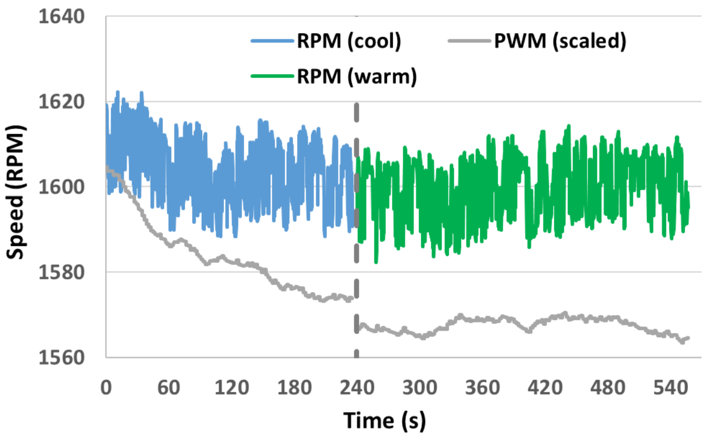

Figure 5: The RPM values in this figure are computed as the median of the 20 most recent RPM measurements. The PWM values are presented in arbitrary units.

SI Figure 5 shows the use of an RP2040 microcontroller running MicroPython to measure tachometer readings and run a *PI* speed control loop. This used a 4 fin tachometer wheel with no rpm values recorded during computationally intensive tasks. The noisy nature of the data requires a slow response to the rpm readings. The PWM signal is 16-bit ($2^{16} = 65536$) resolution, using a P and I of 0.5 and 2 respectively, resulting in an intentionally very slow response. It is noted these P and I values are by coincidence of equal values to those used for the ESP32 based microcontroller. The difference in the resolution makes the values more aggressive for the 13-bit resolution used with the ESP32 based microcontroller.

The system proved usable with the flaw that responsive speed control is not practical for the level of noise in the collected data, allowing only slow correction of the motor speed primarily driven by the I term.

This is a stark contrast to the relatively minimal noise in Figure 10a. The difference between these is suggested a result of how MicroPython handles interrupts on the RP2040 microcontroller, with these being scheduled rather than handled more immediately. The attempts to overcome this problem where not exhaustive, as such solutions may exist. The reasons for moving to the *WEMOS LOLIN32 with OLED* which is an a ESP32 based microcontroller were discussed under the "*Tachometer reading stability*" heading in Section 7.

8. Levich analysis without an electrochemical polishing step

The comparison between using mechanical polishing vs electrochemical polishing as the final step prior to measurements makes a significant difference in the observed currents as presented in SI Figure 4.

This is further extended to show that the surface of the Pt WE has a significant effect on the determined diffusion coefficients. Data with mechanical polishing as the final step is presented in SI Figure 6. The diffusion coefficients were determined as per the methodology in the primary manuscript. This provided values are 6.99e-06 and 7.42e-06 $cm^2\,s^{-1}$ for $Fe(CN)_6{}^{4-}$ and $Fe(CN)_6{}^{3-}$ respectively (R^2 of 0.994 and 0.997). These values are close to the lowest values presented in Table 3 of the manuscript. These values are significantly lower than the diffusion coefficients from the data with electrochemical polishing of 8.95e-06 and 9.88e-06 $cm^2\,s^{-1}$ for $Fe(CN)_6{}^{4-}$ and $Fe(CN)_6{}^{3-}$ respectively (R^2 of 0.9993 and 0.9995 respectively).

The quality of the older data is a representation of the state of the electronics and software used by RDE prototype at that stage of development. The system used MicroPython, and median RPM values were used to exclude the majority of outliers from the displayed RPM readings. The PI control loop had not yet been implemented, as such the RDE required time to warm up while the speed stabilised. Multiple reading from the display were taken down during the measurements to define the error in the RPM values. The speeds were verified by the method in the linked Instructables article (2019) link [1]. This provides a appreciable contrast to the properties and behaviour of the RDE presented in the manuscript.

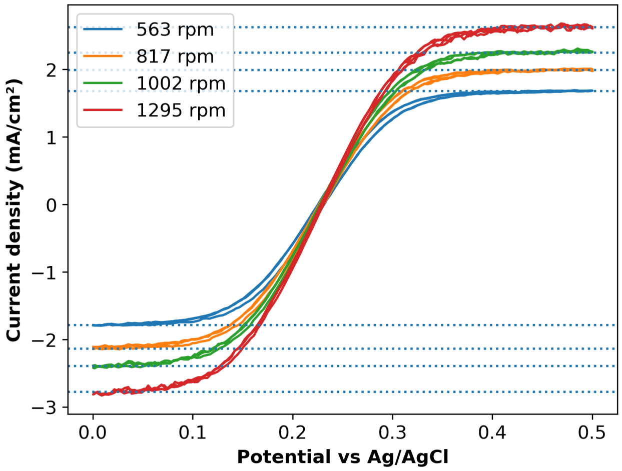

(a)

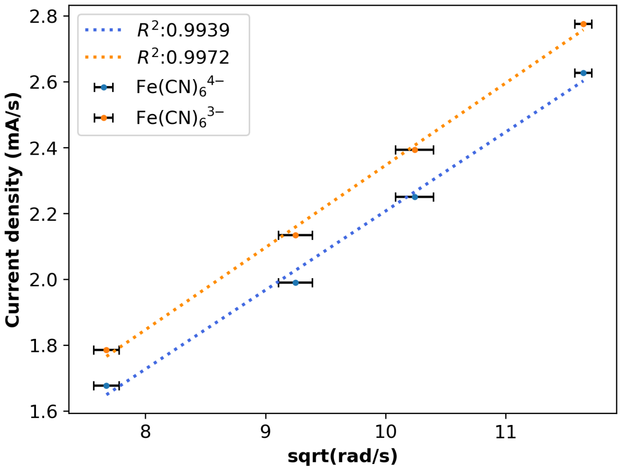

(b)

Figure 6: Electrochemical characterisation of an equimolar 5 mM $K_3[Fe(CN)_6]$, $K_4[Fe(CN)_6] \cdot {}_3H_2O$ solution **without an electrochemical polishing step**. (a) CV with saturation current densities indicated by horizontal lines, (b) Levich plots of the absolute saturation current density for oxidation of $Fe(CN)_6{}^{4-}$ and reduction of $Fe(CN)_6{}^{3-}$ species. *Note: This dataset was collected prior to the introduction of the speed control loop.*

9. Sample loading

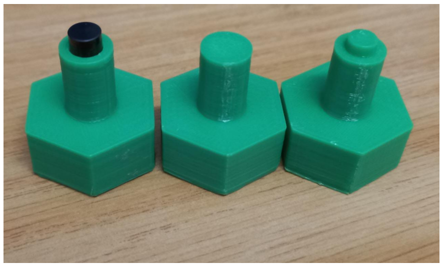

(a)

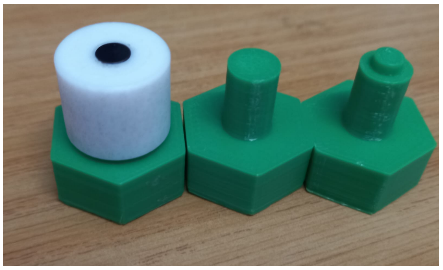

(b)

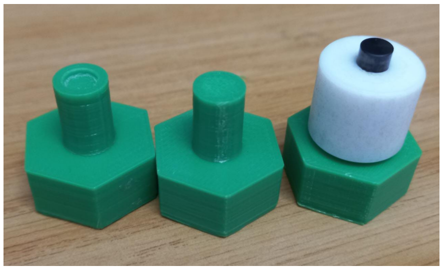

(c)

Figure 7

In using the prototype RDE, an electrode insert is loaded into a “cap”. The insert can be coated prior to loading as required for a given experiment. SI Figure 7 shows the loading and removal process from left to right where a 5 mm glassy carbon electrode is loaded into a “cap”. The tool on the left has a recess to keep the insert centred in place. The middle tool is used to push the insert deeper into the “cap”, with SI Figure 7c showing removal of the insert.

10. 3D Printer settings

Prints were made with either PETG or ABS filaments. For small parts where a M3 or M4 thread is present, parts for the rotor were printed with 30 % fill, 0.1 mm resolution and a raft with the exception of the *cap base* which was not printed with a raft.

The mounts and bearing covers were printed with a 30 % fill, 0.15 mm resolution and no raft. The stand components were printed in solid ABS with a 0.15mm resolution.

The components were printed with a Wanhao 5S mini using 2.85 mm filament. While the printer is specified as using 3.0 mm, the narrower filament achieve more accurate print tolerances. A test attempting to reduce the filament feed rate to 50 % using the Wanhao Maker software appeared ineffective on the end result. It suggested either the feed rate could not be adjusted on the specific printer or the tested value may have been

out of range of what the printer accepts.

The printer was equipped with an aftermarket heated borosilicate glass print-bed. Masking tape was used to cover the print bed with hairline gaps between the strips. For ABS prints, a solution of ABS in acetone was spread over the print area for improved adhesion.

The print space was partially enclosed with acrylic panels with large openings at the top where the cable and filament feed into the extruder, as well as the side for the cable connected to the hotbed. For the *rotor mount* and *cap base* printed in ABS, a draft shield was printed surrounding the part with the intention of reducing drafts and warping, Figure 8.

ABS and PETG have similar thermal expansion coefficients of 7.2×10^{-5} and 7×10^{-5} m/(m K) respectively [2]. When considered with the higher print temperatures of ABS this translates to more shrinkage for the material on cooling. In addition to this ABS is well documented as more susceptible to warping [3, 4].

Table 1: 3D Printer settings

Polymer	Nozzle temp. (°C)	Bed temp. (°C)	Print speed (mm/s)	Fan (%)
PETG	225	75	50	100
ABS	235	95	50	0

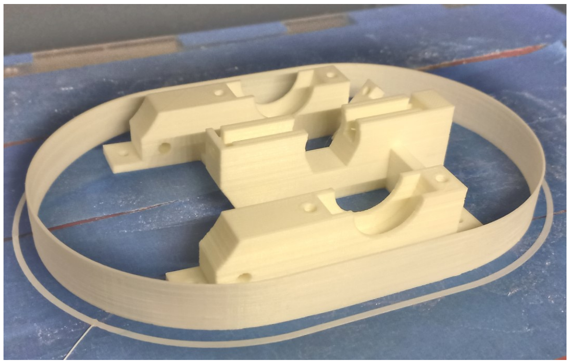

(a)

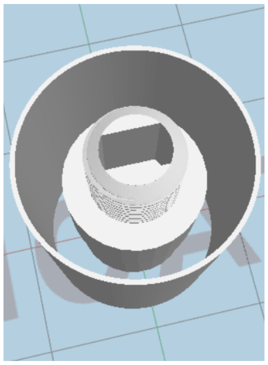

(b)

Figure 8: (a) Printed *rotor mount* on printer. (b) Modelled *cap base* for printing.

11. Design file descriptions (full list)

- *Shnier 2024-RDE-SI.pdf* includes additional detail and supporting information on the fabrication, validation and design of the presented prototype.
- *Clip_and_height_adjustment.mp4*, a video showing the height adjustment mechanism for the RDE's stand.
- *Operation_and_adjustment_(20240724).mp4*, showing a full view of the RDE and the operation of the RDE and adjustment of speed.
- *RDE_Main_r0.6.8_GJbrushes_wider.FCStd*, The FreeCAD design file containing the *motor mount*, *rotor mount*, GT2 driven pulley, tachometer wheel, bearing covers and brush holder clip. This variant was used for collection of the results in Section 7.
- *RDE_Main_r0.7.0_shorter-length.FCStd*, The FreeCAD design file containing the same components as the above entry. This variant is more compact then the variant marked "wider".
- *RDE_GT2_driven_pulley.FCStd*, This is a FreeCAD design file for the driven pulley.
  - The r0 version of this part was used for RDE Versions HX1 and HX2. An proposed updated version, r1 is included. The updated version moved the M3 grub screw away from the GT2 teeth and provides 4 threaded holes rather than 1. Only two opposing holes are intended for use. The remaining 2 available as spare in case of a print defect.
- *RDE_motor_GT2_driving_pulley_20tooth_6p65_hole.FCStd*, This is a FreeCAD design file for the driving pulley.

- *RDE_tachometer_wheel_4fin.FCStd* and variants, These are a FreeCAD design files for the tachometer wheel.
- *RDE_cap_base (two varients)*, The FreeCAD design file for the *cap base*, specifically *RDE_cap_base_r0.4.4_PETG.FCStd* and *RDE_cap_base_r0.5.0_ABS.FCStd*. This part is press fit into the end of the 15 mm copper tube. It hold the sprung test probe and the *cap* into which the sample inserts are fitted. The *r0.4.4_PETG* version was used for the results in Section 7.
- *RDE_cap_r0.4_16mm-dia_14mm_5p2bore_025clear*, The FreeCAD design file for the *cap* which holds a 5 mm diameter, 4 mm height insert to serve as a working electrode.
- *Electronics_clip (two variants*, These are *Electronics_clip_2ndBox_r1.FCStd* and *Electronics_clip_2ndBox_r1_tapered.* The FreeCAD design files for the clips used to keep the PCB in place inside the electronics box.
- *M3_nut.FCStd*, the FreeCAD design file for a basic M3 nut.
- (Folder) RDE stand (FreeCAD and STL), the FreeCAD design files and STL meshes for the stand portion of the RDE.
  - *Stand_attach*, this screws onto the wooden board and accommodates the 8 mm stainless steel (SS) bars on which the RDE slides.
  - *Stand_bottom*, this is a support that connects the base of the stand to the 8 mm SS bars using four M3 grub screws. The majority of the support comes from holes drilled into the base of the stand.
  - *Stand_stop*, this holds itself onto the SS bars to set the height of the RDE. It is fastened using an M6 bolt and wingnut.
  - *Stand_hold*, this holds the RDE onto the SS bars. Once the desired height is set with the *stand stop*, this allows the user to raise the RDE and hold it securely in place to change samples without moving the *stand stop*. It is fastened using an M6 bolt, M6 nut and a M6 wingnut.
  - *Stand_top*, this holds the SS bars at their heighest point, adding rigidity to stand. This is fastened to the SS bard using four M3 grub screws.
- *(Folder) Electrode insert mounting set*, A set of FreeCAD files of tools for loading 5 mm diameter electrode inserts into a *cap*.
- *STL files for 3D printing*, these files are available in the Mendeley data repository. They have not been individually listed to avoid duplication of the FreeCAD files from which they were exported.
- *RDE_code.ino (v0.1)*, the Arduino code for the ESP32 based microcontroller. This is available on both GitHub and the Menedeley data repository.

12. Replacing a sprung test probe or cap base

The prototype presented is being using in a corrosive, alkaline environment. While care can be taken to wash the probe should a leak occur or if the electrode insert is not properly fitted in the *caps*, the sprung probes and/or PETG *cap base* can deteriorate. The failure mode of the cap base is for the thread to break after repeated exposure to strongly alkaline liquid. In normal operation, the *cap base* does not come into any contact with strongly alkaline liquid.

P.1 Pull the sprung test probe out of the *cap base* with tweezers or small pliers.

P.2 (optional) Remove some of the solder using a soldering iron and de-soldering pump.

P.3 Heat the soldered joint and unroll the probe by pushing it away from the wire using tweezers held open against one side of the probe, symmetrically across the wire.

P.4 If the *cap base* requires replacing this can be done now.

- The copper can be wiped down with a dilute acid followed by water to remove any corrosion, this can be done at any time as required. When the *cap base* is present the RDE should be orientated such that the acid does not run towards the *cap base* to which it could carry contaminants.

P.5 The wire can be cleaned of solder or the solder can be moved with the soldering iron to make it easier to wrap the wire around the probe.

P.6 The probe can be soldered and fitted as described in the previous section.

13. Failed searches for 12V DC motors with a built in encoder or sensor

The following list presents search terms used across multiple platforms for a suitable motor for the rotating disk electrode prototype with a build in speed sensor or encoder. While not exhaustive, the results a suitable motor with the following properties was not found, DC motors that can be operated by a single PWM signal, operating voltage below 24 V, reaching $\geq$ 3000 rpm, with a built in speed sensor, and cost below 100 USD. These searches were made from a South African IP address on 1 May 2024 unless otherwise stated. The use of a brushless DC (BLDC) motor is considered in SI Section 14.

Google Shopping, search terms attempted

- "12 V DC motor encoder"
- "DC motor encoder"
- "dc motor hall sensor"
- "DC motor sensor"

Amazon, search terms attempted

- "12 V DC motor with in-built rotary encoder"
- "DC motor with encoder"
- "DC motor with encoder 10000rpm"

Micro Robotics, search terms attempted (South African company)

- "motor encoder"
- "motor sensor"
- "bldc" (accessed 21 June 2024)

Mantech, search terms attempted (South African company)

- "motor hall"
- "motor sensor"
- "bldc" (accessed 21 June 2024)

14. Three phase motor option

A 3-phase motor and controller set that was close to the desired specification was found between Mantech.co.za and Micro Robotics (Part No. 35ZWN24-20, Product code: MRX-BL4805F) with a rated speed of 3000 rpm and a no-load speed of 4500 rpm at 24 V. It is noted that 3-phase brushless DC (BLDC) motors were not considered during the design process, these may be suitable, with the compromise of requiring a dedicated motor controller adding cost to the design. The sensor output may then needed to be converted to from 5 to 3.3 V logic levels which can be facilitate using a TXS0108 logic level converter.

References

[1] A. Shnier, Spin coater v1 (almost analog), (accessed: 2024-07-23) (2019).
URL https://www.instructables.com/Spin-Coater-V1-almost-Analog/

[2] B. Rădulescu, A. M. Mihalache, A. Hrițuc, M. Rădulescu, L. Slătineanu, A. Munteanu, O. Dodun, G. Nagîț, Thermal expansion of plastics used for 3d printing, Polymers 14 (15) (2022). doi:10.3390/polym14153061.

[3] G. Holcomb, E. B. Caldona, X. Cheng, R. C. Advincula, On the optimized 3d printing and post-processing of petg materials, MRS Commun. 12 (3) (2022) 381–387. doi:10.1557/s43579-022-00188-3.

[4] J. Ramian, J. Ramian, D. Dziob, Thermal deformations of thermoplast during 3d printing: warping in the case of abs, Materials 14 (22) (2021) 7070. doi:10.3390/ma14227070.
